# Targeting fibroblast activation protein (FAP): next generation PET radiotracers using squaramide coupled bifunctional DOTA and DATA^5m^ chelators

**DOI:** 10.1186/s41181-020-00102-z

**Published:** 2020-07-29

**Authors:** Euy Sung Moon, Filipe Elvas, Gwendolyn Vliegen, Stef De Lombaerde, Christel Vangestel, Sven De Bruycker, An Bracke, Elisabeth Eppard, Lukas Greifenstein, Benedikt Klasen, Vasko Kramer, Steven Staelens, Ingrid De Meester, Pieter Van der Veken, Frank Rösch

**Affiliations:** 1 Department of Chemistry – TRIGA Site, Johannes Gutenberg University Mainz, 55128 Mainz, Germany; 2 Department of Nuclear Medicine, Antwerp University Hospital (UZA), 2650 Edegem, Belgium; 3 Department of Pharmaceutical Sciences, Laboratory of Medical Biochemistry, University of Antwerp, 2610 Wilrijk, Belgium; 4 Molecular Imaging Center Antwerp (MICA), University of Antwerp, 2610 Wilrijk, Belgium; 5 Positronpharma SA, 7500921 Providencia, Santiago, Chile

**Keywords:** DOTA, DATA^5m^, Gallium-68, FAP, PREP, Squaric acid, Squaramide, HT-29

## Abstract

**Background:**

Fibroblast activation protein (FAP) is a proline selective serine protease that is overexpressed in tumor stroma and in lesions of many other diseases that are characterized by tissue remodeling. In 2014, a most potent FAP-inhibitor (referred to as UAMC1110) with low nanomolar FAP-affinity and high selectivity toward related enzymes such as prolyl oligopeptidase (PREP) and the dipeptidyl-peptidases (DPPs): DPP4, DPP8/9 and DPP2 were developed. This inhibitor has been adopted recently by other groups to create radiopharmaceuticals by coupling bifunctional chelator-linker systems. Here, we report squaric acid (SA) containing bifunctional DATA^5m^ and DOTA chelators based on UAMC1110 as pharmacophor. The novel radiopharmaceuticals DOTA.SA.FAPi and DATA^5m^.SA.FAPi with their non-radioactive derivatives were characterized for in vitro inhibitory efficiency to FAP and PREP, respectively and radiochemical investigated with gallium-68. Further, first proof-of-concept in vivo animal study followed by ex vivo biodistribution were determined with [^68^Ga]Ga-DOTA.SA.FAPi.

**Results:**

[^68^Ga]Ga-DOTA.SA.FAPi and [^68^Ga]Ga-DATA^5m^.SA.FAPi showed high complexation > 97% radiochemical yields after already 10 min and high stability over a period of 2 h. Affinity to FAP of DOTA.SA.FAPi and DATA^5m^.SA.FAPi and its ^nat^Ga and ^nat^Lu-labeled derivatives were excellent resulting in low nanomolar IC_50_ values of 0.7–1.4 nM. Additionally, all five compounds showed low affinity for the related protease PREP (high IC_50_ with 1.7–8.7 μM). First proof-of-principle in vivo PET-imaging animal studies of the [^68^Ga]Ga-DOTA.SA.FAPi precursor in a HT-29 human colorectal cancer xenograft mouse model indicated promising results with high accumulation in tumor (SUV_mean_ of 0.75) and low background signal. Ex vivo biodistribution showed highest uptake in tumor (5.2%ID/g) at 60 min post injection with overall low uptake in healthy tissues.

**Conclusion:**

In this work, novel PET radiotracers targeting fibroblast activation protein were synthesized and biochemically investigated. Critical substructures of the novel compounds are a squaramide linker unit derived from the basic motif of squaric acid, DOTA and DATA^5m^ bifunctional chelators and a FAP-targeting moiety. In conclusion, these new FAP-ligands appear promising, both for further research and development as well as for first human application.

## Background

The proline-selective serine protease fibroblast activation protein (FAP) is a type II transmembrane glycoprotein with 760 amino acids. It is related to the dipeptidyl peptidases (DPPs) DPP2, DPP4, DPP8 and DPP9 and furthermore related to the endopeptidase prolyl oligopeptidase (PREP) (Brennen et al. 2012; Dvořáková et al. 2017). FAP combines DPP and endopeptidase activities (Aertgeerts et al. 2005; Edosada et al. 2006; Levy et al. 1999; Park et al. 1999). With respect to FAP’s endopeptidase activity, a remarkable preference is present for cleavage after Gly-Pro motifs in peptides (Bracke et al. 2019). FAP is not detectable in most healthy adult tissues and therefore considered non-essential under normal circumstances. However, it is clearly expressed in pathophysiological lesions, characterized by tissue remodeling. Such lesions can be found in, e.g., cancer, atherosclerosis, arthritis and several fibrosis types (Hamson et al. 2014; Liu et al. 2012). Over the past two decades, significant attention has gone to FAP in solid tumors, where it is mainly expressed on so-called cancer associated fibroblasts (CAFs) (Chen and Song 2019; Jiang et al. 2016). These are activated fibroblasts with a myofibroblast phenotype (Liu et al. 2019). There is growing evidence that CAFs have a regulatory role in tumor biology and extracellular matrix composition (Tao et al. 2017; De Vlieghere et al. 2015; Zi et al. 2015). FAP^+^-CAFs are present in the stromal tissue of more than 90% of epithelial carcinomas, including pancreatic, colon, ovarian, lung and breast cancer (Busek et al. 2018; Scanlan et al. 1994). Generally speaking, the tumor stroma contains a large part of the tumor mass (> 90% of tumor mass in carcinomas) and is therefore an attractive target for diagnostic and therapeutic radiopharmaceuticals. Conferring stroma affinity to these radiopharmaceuticals by incorporating a FAP-inhibitor moiety is, based on the presence of FAP^+^-CAFs, a potentially rewarding strategy.

**Fig. 1:**
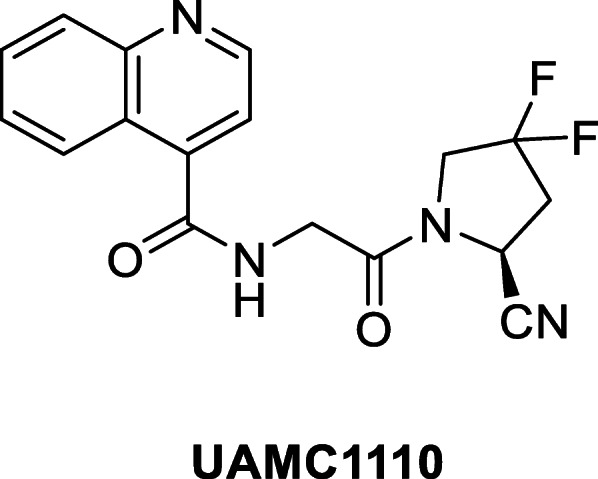
FAP-inhibitor lead structure UAMC 1110

Several highly potent FAP-inhibitors have been reported earlier (Connolly et al. 2008; Poplawski et al. 2013). First-generation compounds with a boronic acid warhead, however, are plagued by a lack of selectivity with respect to the related enzymes and are also characterized by lower chemical stability. More recently, compounds with a more stable carbonitrile warhead have been reported (Jansen et al. 2014b; Jansen et al. 2014a). One of the most promising molecules to date is UAMC1110 (Fig. 1). This molecule combines low nanomolar FAP affinity and high selectivity with respect to both the DPPs and PREP. The high FAP-selectivity of UAMC1110 is particularly attractive for tumor-targeting, when taking into account the near-ubiquitous expression of the DPPs and PREP in humans. In addition, this molecule possesses a satisfactory pharmacokinetic profile.

UAMC1110 is currently still under evaluation as a potential therapeutic in diseases characterized by FAP expression. At the same time, the molecule is being used as a FAP-targeting moiety in so-called activity-based probes that can be used to visualize and quantify FAP activity in tissues and organisms (De Decker et al. 2019). Highly relevant examples have also been published that rely on radionuclide-based reporter systems, such as XY-FAP-02 developed by Yang et al. (Yang et al. 2019)*.* They used a DOTAGA chelator combined with an alkyl chain as linker system bound to the FAP-inhibitor.

Further development of radiotracers by Lindner and Loktev et al. based on the FAP inhibitors from Antwerp have shown promising results in preclinical and first clinical patient studies. Applications of these molecules cover both diagnosis and therapy (Giesel et al. 2019b; Giesel et al. 2019a; Kratochwil et al. 2019; Lindner et al. 2018; Loktev et al. 2019; Loktev et al. 2018). First, a DOTA-FAPI conjugate using piperazine as linker (referred to as FAPI-02 in the original reference) was synthesized and characterized with respect to binding, internalization, and efflux in cells expressing human and murine FAP as well as CD26. PET-imaging studies of HT-1080 tumor xenografts showed low [^68^Ga]Ga-FAPI-02 accumulation in normal tissues and a rapid clearance from the blood via kidneys and bladder. In addition, a high tumor uptake resulting in high tumor-to-normal organ-ratio was determined. By structural variation, especially in the linker region, more analogous gallium-68 labeled compounds were obtained. Several of these had improved imaging parameters, with FAPI-04, FAPI-21 and FAPI-46 being relevant examples (Lindner et al. 2018; Loktev et al. 2019). These compounds also had low nanomolar FAP-affinities, higher tumor uptakes in vivo and longer tumor retention times. First PET/CT imaging studies of patients diagnosed with different tumor entities were performed with the gallium-68 compounds indicating high tumor uptake and low background in healthy organs. As an example of a first therapeutic application, patients diagnosed with metastatic breast cancer were treated with [^90^Y]Y-FAPI-04. The ^68^Ga/^90^Y-DOTA-derivatives represent promising tracers for both diagnostic imaging and, possibly, targeted therapy of malignant tumors with high accumulated activated fibroblasts.

In this work, novel FAP-targeting radiotracers were evaluated using bifunctional DOTA and DATA^5m^ chelators coupled by squaramide as linker moiety. The basic motif squaric acid (SA) is a cyclic aromatic diacid (Ian Storer et al. 2011; Wurm and Klok 2013). One advantage of SA is the simple chemistry regarding coupling to chelator and target vector including that no protecting groups are necessary due to its selectivity for primary amines. Especially reactions with biomolecules are attractive and no side reactions are observed. The coupling with SA-diester is a highly selective, pH controlled asymmetric amidation under mild conditions (Tietze et al. 1991). In a neutral pH, only one ester of the SA-diester reacts with an amine and by increasing the pH to basic conditions, amidation of the second ester takes place. The use of SA as a linker unit between a chelator-biomolecule conjugate as a radiopharmaceutical was demonstrated using DFO and conjugation on a peptide to complex iron and using DFO-squaric acid coupled to antibodies for complexing zirconium-89 (Rudd et al. 2016; Yoganathan et al. 2011). Recently, our group published the usage of SA as a linker forming a radiotracer with the bifunctional hybrid chelator AAZTA^5^ coupled to a PSMA inhibitor unit (KuE) and evaluated those AAZTA^5^.SA.PSMA conjugate with various radionuclides such as scandium-44, copper-64, gallium-68 and lutetium-177 (Greifenstein et al. 2019). Additionally, we indicate a second feature of SA beyond coupling chemistry. In several cases we could observe a positive impact on pharmacology of the final products. [^68^Ga]Ga-NODAGA.SA.PSMA, [^68^Ga]Ga-TRAM.SA.PSMA and [^68^Ga]Ga-DOTAGA.SA.PSMA showed high tumor uptake and overall high tumor-to-organ ratio. [^68^Ga]Ga-DOTAGA.SA.PSMA provided in vivo in LNCaP-tumor bearing mice comparable results to [^68^Ga]Ga-PSMA-617 and [^68^Ga]Ga-PSMA-11 with significant tumor accumulation (Greifenstein et al. 2020).

**Fig. 2:**
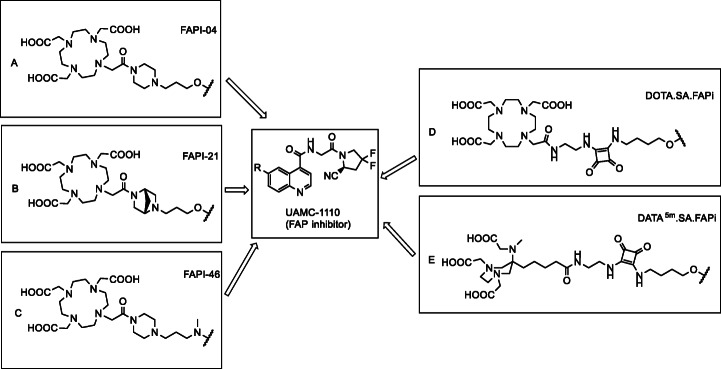
Structural comparison of chelator-linker conjugates coupled with UAMC1110 (FAP inhibitor). **a** FAPI-04; **b** FAPI-21; **c** FAPI-46; **d** DOTA.SA.FAPi; **e** DATA^5m^.SA.FAPi

Here, the preparative synthesis of DOTA.SA.FAPi and DATA^5m^.SA.FAPi and the metal-analogs [^nat^Ga]Ga-DOTA.SA.FAPi, [^nat^Ga]Ga-DATA^5m^.SA.FAPi and [^nat^Lu]Lu-DOTA.SA.FAPi are described. The macrocyclic chelator DOTA was used to allow labeling with both gallium-68 and lutetium-177. However, one disadvantage of these chelator types are the requirement of high temperatures for complexation (Price and Orvig 2014). DATA^5m^, a bifunctional version of the hybrid chelator DATA, was used to allow instant gallium-68 labeling at room temperature (Seemann et al. 2017; Seemann et al. 2015; Sinnes et al. 2019). Radiochemical evaluation with regard to labeling and in vitro stability studies were performed with gallium-68 for DOTA.SA.FAPi and DATA^5m^.SA.FAPi. For all the five cold compounds, inhibition assays were carried out and IC_50_ values obtained for FAP and PREP. In a first proof-of-principle PET-study, [^68^Ga]Ga-DOTA.SA.FAPi was tested in vivo using a FAP-expressing HT-29 human colorectal adenocarcinoma xenograft model. Figure 2 shows the squaric acid containing tracers DOTA.SA.FAPi and DATA^5m^ .SA.FAPi based on UAMC1110.

## Results

### Synthesis of DOTA.SA.FAPi

**Fig. 3:**
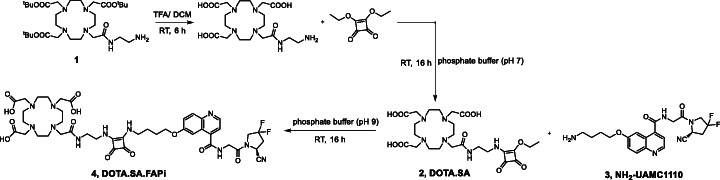
Synthesis scheme of DOTA.SA.FAPi 4

The commercially available DO3A*t*Bu-*N*-(2-aminoethyl) ethanamide **1** was treated with TFA to deprotect the *tert*-butyl groups. Since the coupling of squaric acid diethyl ester (SADE) with primary amines is selective, no protective groups were necessary for the next synthesis steps. The deprotected DO3A-*N*-(2-aminoethyl) ethanamide was coupled to SADE in phosphate buffer (pH 7) at ambient temperature and purified via HPLC to receive DOTA.SA **2**. The free coupling side of **2** was afterwards coupled to (*S*)-6-(4-aminobutoxy)-*N*-(2-(2-cyano-4,4-difluoropyrrolidin-1-yl)-2-oxoethyl)-quinoline-4-carboxamide **3** (termed NH_2_-UAMC1110) in phosphate buffer (pH 9) at room temperature. After successful HPLC purification, DOTA.SA.FAPi **4** was obtained (Fig. 3).

### Synthesis of DATA^5m^.SA.FAPi

**Fig. 4:**
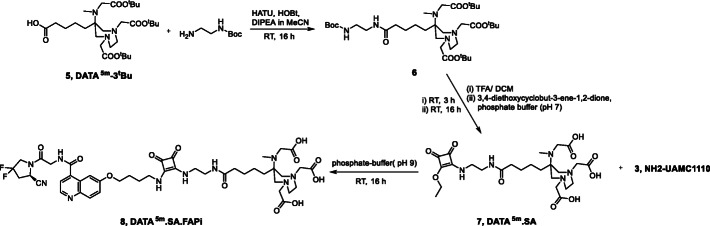
Synthesis scheme of DATA^5m^.SA.FAPi 8

DATA^5m^-3^t^Bu **5** was synthesized as described by Seemann et al. (Fig. 4) (Seemann et al. 2017). DATA^5m^-3^t^Bu provides a bifunctional carbonyl group for further coupling with spacer molecules or target vectors. Terminal primary amines are required for binding to SA-diethylester. Therefore *N*-boc-ethylenediamine was attached to the carboxylic acid group of DATA^5m^ via common coupling reagents HATU in DIPEA and acetonitrile receiving **6**. Amidation of SA-monoester **7** with the terminal amine of NH_2_-UAMC1110 was executed analogously to DOTA.SA.FAPi to receive DATA^5m^.SA.FAPi **8**.

### Synthesis of cold complexes and enzyme inhibition assays

Non-radioactive complexes of [^nat^Ga]Ga-DOTA.SA.FAPi, [^nat^Ga]Ga-DATA^5m^.SA.FAPi and [^nat^Lu]Lu-DOTA.SA.FAPi were synthesized. The corresponding precursors were reacted with a 10 mM solution of the metal chlorides or nitrates in 0.2 M natrium acetate (NaAc) buffer pH 4.5. The solutions of ^nat^Ga and ^nat^Lu complexed DOTA.SA.FAPi were shaken for 3 h at 95 °C and the solution of ^nat^Ga-metalled DATA^5m^.SA.FAPi was shaken for 2 h at RT. Complexations were monitored by ESI LC-MS and the metal complexes were purified via HPLC.

In the inhibition assays, DOTA.SA.FAPi and DATA^5m^.SA.FAPi, along with their non-radioactive, metal complexed analogues were characterized for inhibitory potency towards FAP and PREP. Earlier work had shown that the lack of a basic amine function in UAMC1110-based molecules, precludes DPP-affinity in this series (De Decker et al. 2019; Jansen et al. 2014a). Nonetheless, the FAP/PREP selectivity was shown to be a particularly important parameter to check. Obtained results are summarized in Table 1. Parent compound UAMC1110 was used as a reference in this assay. All the evaluated molecules displayed highly satisfactory, low nanomolar FAP potencies, in the same range as the parent inhibitor UAMC1110. This implies that introduction of a linker, a chelator and a metal ion at the selected position of the quinoline ring are tolerated by FAP and have no negative influence on target affinity. Likewise, equally satisfactory compound selectivities with respect to PREP were measured, again comparable with UAMC1110.

**Table 1:** IC_50_-values of DOTA.SA.FAPi, the ^nat^Ga and ^nat^Lu-complexes and DATA^5m^.SA.FAPi and the ^nat^Ga-complex with regard to FAP and PREP. Selectivity index gives the ratio FAP to PREP

	IC_50_ FAP (nM)	IC_50_ PREP (μM)	Selectivity index (IC_50_ (FAP/PREP))
DOTA.SA.FAPi – uncomplexed	0.9 ± 0.1	5.4 ± 0.3	6000
DOTA.SA.FAPi - ^nat^Ga	1.4 ± 0.2	8.7 ± 0.9	6214
DOTA.SA.FAPi – ^nat^Lu	0.8 ± 0.2	2.5 ± 0.4	3125
DATA^5m^.SA.FAPi – uncomplexed	0.8 ± 0.2	1.7 ± 0.1	2113
DATA^5m^.SA.FAPi – ^nat^Ga	0.7 ± 0.1	4.7 ± 0.3	6714
FAP-inhibitor UAMC1110	0.43 ± 0.07^a^	1.8 ± 0.2^b^	4186

^a^Determined under the conditions of this study. ^b^ data from Jansen et al. (Jansen et al. 2014a)

### Radiochemical evaluations with gallium-68

**Fig. 5:**
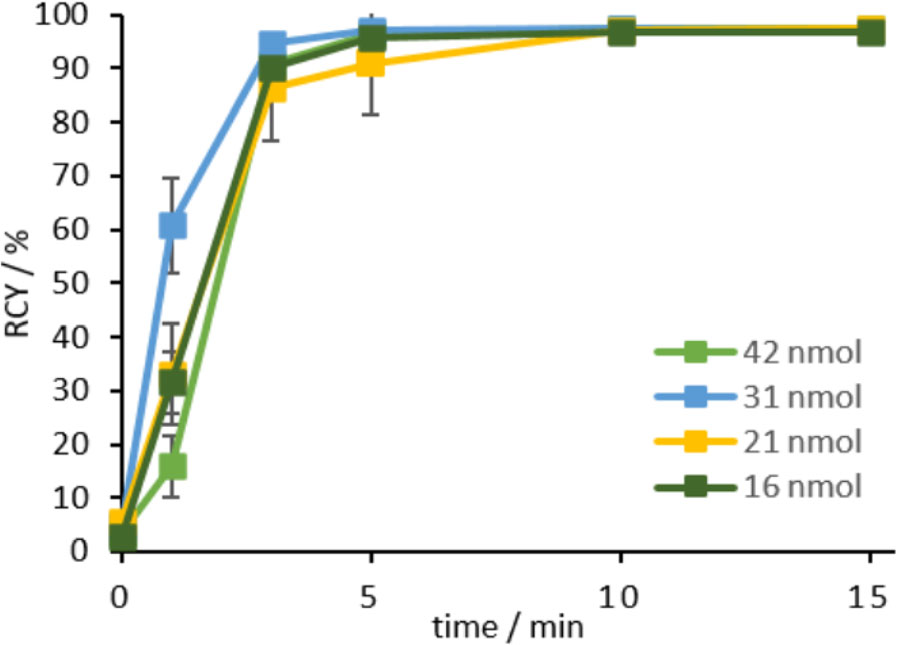
Radiolabeling kinetics for various amounts of [^68^Ga]Ga-DOTA.SA.FAPi complex at 95 °C, precursor amounts > 15 nmol result in RCY > 97% after 15 min

Radiolabeling of DOTA.SA.FAPi with ^68^Ga was performed with varying amounts of the precursor (11–42 nmol) and at 95 °C (Fig. 5). Labeling was performed in 300 μl 1 M ammonium acetate (AmAc) buffer (pH 5.5) at 95 °C in triplicate *n* = 3 with around 200 MBq of gallium-68. For precursor amounts of more than 16 nmol, a quantitative radiochemical yield (RCY) of > 97% could be achieved in less than 5 min. At 11 nmol a decreased RCY of 44% after 15 min could be observed. HPLC retention time of free gallium-68: *t*_*R*_ (^68^Ga) = 4 min and the retention time of the complex *t*_*R*_ ([^68^Ga]Ga-DOTA.SA.FAPi) = 9 min. The *R*_f_ values of the radio-TLC were *R*_*f*_ (^68^Ga) = 0.9 and *R*_*f*_ ([^68^Ga]Ga-DOTA.SA.FAPi) = 0.1 using citrate buffer pH 4 as mobile phase.

Carrying out labeling of DOTA.SA.FAPi at different temperatures (70, 80 and 95 °C) with a defined precursor amount of 31 nmol resulted in quantitative RCYs > 97% at temperatures of 80 °C and 95 °C after 15 min. At 70 °C, complexation of gallium-68 via DOTA.SA.FAPi showed decreased radiolabeling efficiency, nevertheless resulting in > 83% after 15 min. (SI, Fig. S3).

Stability studies were performed in ethanol (EtOH), human serum (HS) and saline 0.9% (NaCl) over a period of 2 h at 37 °C. In all three media, [^68^Ga]Ga-DOTA.SA.FAPi showed high stabilities over 98% intact conjugate (SI, Fig. S4). In addition, stability against transmetallation and transchelation were carried out (SI, Fig. S5, S6). Against DTPA and EDTA the stability values were > 98% and against Cu, Mg and Ca the stabilities were > 95% after 2 h. Stabilities against Fe showed > 95% after 90 min and a slightly lower value however still over 92% after 2 h.

**Fig. 6:**
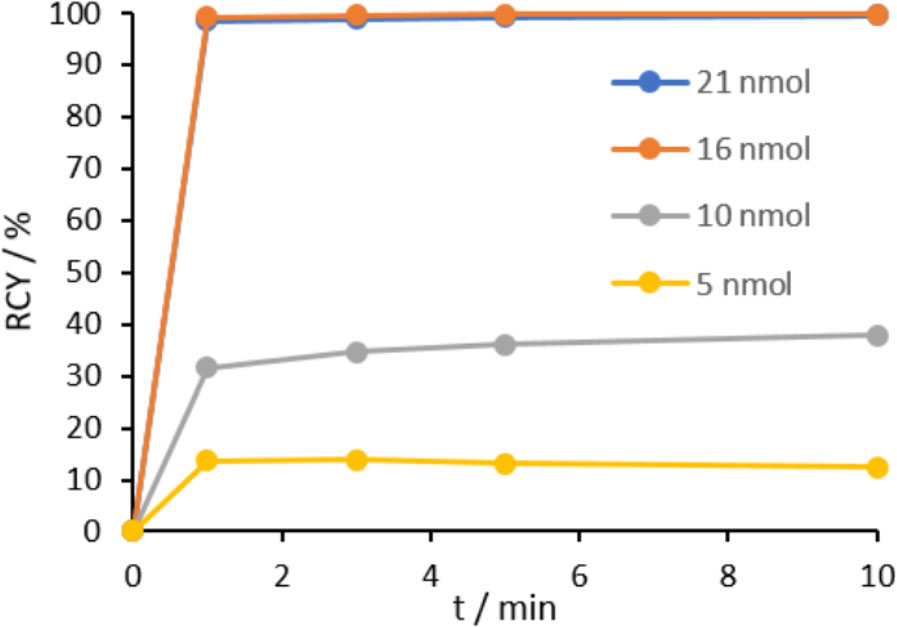
Radiolabeling kinetics of various amounts of [^68^Ga]Ga-DATA^5m^.SA.FAPi complex at RT, precursor amounts > 15 nmol result in RCY > 98% after 15 min

For radiolabeling of DATA^5m^.SA.FAPi, various precursor amounts (1–21 nmol) in 300 μl 1 M AmAc buffer (pH 5.5) were labeled with gallium-68 (Fig. 6). The reaction mixture was shaken for 10 min at room temperature to afford [^68^Ga]Ga-DATA^5m^.SA.FAPi.

First kinetic studies were performed with 200–230 MBq of gallium-68 in 1 M AmAc buffer (pH 5.5) at RT (*n* = 1). Quantitative RCYs > 98% could be achieved for precursor amounts of 16 nmol and 21 nmol in less than 1 min. Analogous to [^68^Ga]Ga-DOTA.SA.FAPi, [^68^Ga]Ga-DATA^5m^.SA.FAPi also showed significantly lower RCY below 15 nmol. At 10 nmol only a RCY of 38% and for 5 nmol a RCY of just 12% after 10 min could be observed. HPLC retention times of free gallium-68 *t*_*R*_ (^68^Ga) and of the complex *t*_*R*_ ([^68^Ga]Ga-DATA^5m^.SA.FAPi) were 4 min and 8.5 min, respectively. The *R*_f_ values of the radio-TLC were *R*_*f*_ (^68^Ga) = 0.9 and *R*_*f*_ ([^68^Ga]Ga-DATA^5m^.SA.FAPi) = 0.1 using citrate buffer pH 4 as mobile phase. Stability of [^68^Ga]Ga-DATA^5m^.SA.FAPi was determined in phosphate buffered saline (PBS), HS and NaCl over a period of 120 min. In all three media, the stability of [^68^Ga]Ga-DATA^5m^.SA.FAPi remained > 95% (SI, Fig. S7).

### PET/CT-imaging and ex vivo biodistribution data of [^68^Ga]Ga-DOTA.SA.FAPi

**Fig. 7:**
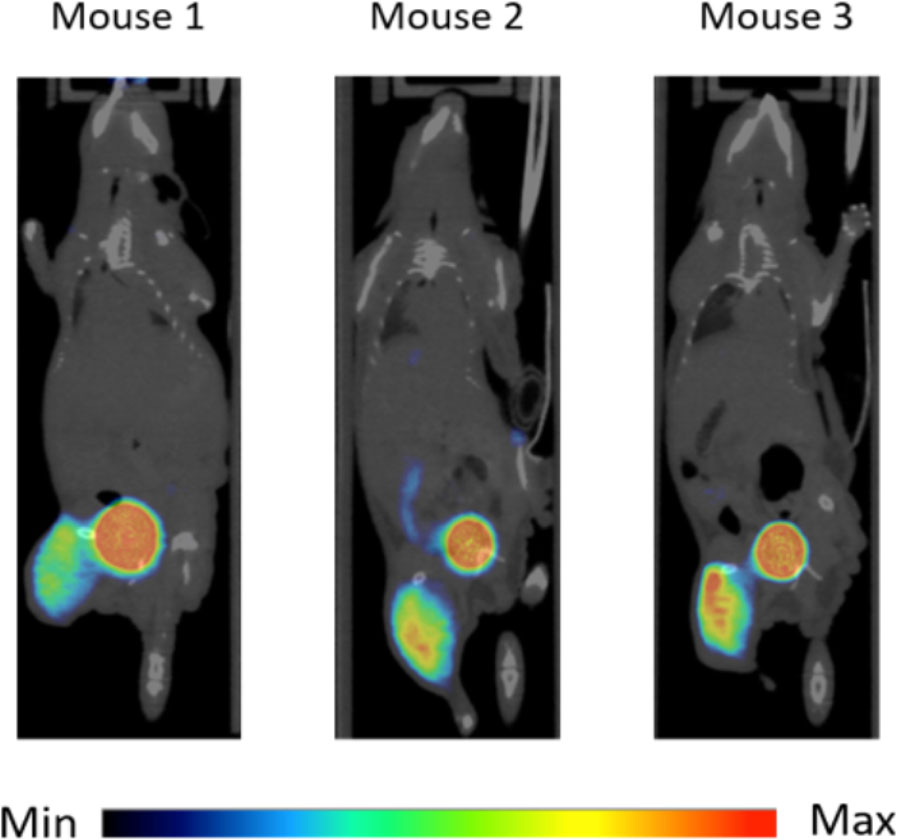
In vivo evaluation of [^68^Ga]Ga-DOTA.SA.FAPi uptake in a HT-29 xenograft mouse model. Representative coronal small-animal PET/CT images (MIP) 60 min after injection of [^68^Ga]Ga-DOTA.SA.FAPi

**Fig. 8:**
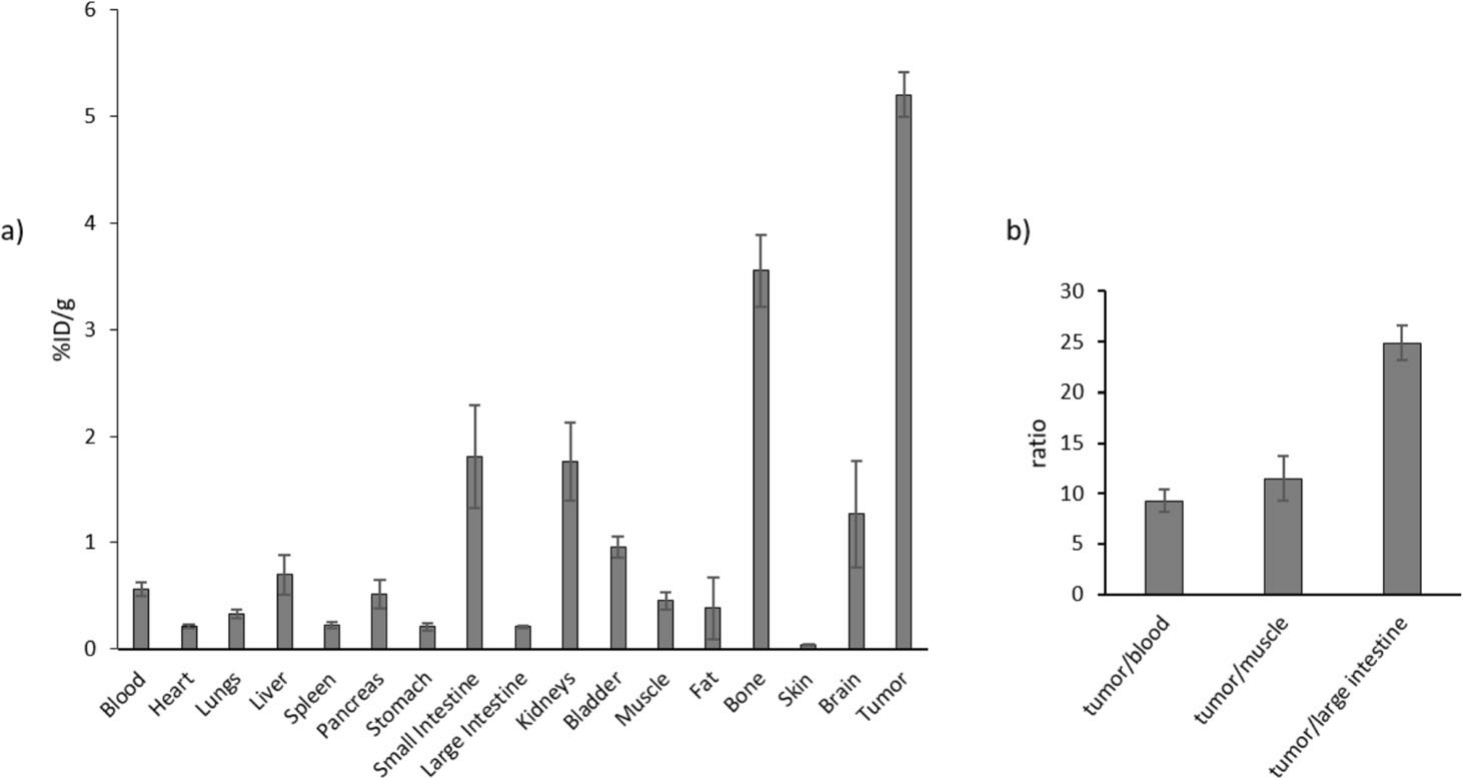
**a**) Ex vivo biodistribution of [^68^Ga]Ga-DOTA.SA.FAPi in HT-29 xenograft mice (*n* = 3) 1 h p.i. **b**) Tumor-to-organ ratios of [^68^Ga]Ga-DOTA.SA.FAPi in HT-29 xenograft mice (*n* = 3) 1 h p.i

For investigation of the tumor uptake by [^68^Ga]Ga-DOTA.SA.FAPi HT-29 tumor-bearing mice (*n* = 3) were sacrificed after PET/CT scans and an ex vivo biodistribution study was executed. In the PET images, the tumor accumulation is clearly visible (SUV_mean_ of 0.75 ± 0.09) and the ratio to nonspecific organs and tissues is very high (SUV_mean_: 0.15 ± 0.01 in the heart, 0.18 ± 0.07 in the muscle, 0.37 ± 0.14 in the small intestine, 0.27 ± 0.11 in kidneys and 0.22 ± 0.08 in the liver)). Figure 7 shows the maximum intensity projection (MIP) images of three mice. Ex vivo biodistribution is shown in Fig. 8a. The accumulation in the tumor at 60 min post injection (p.i.) as found in both the PET images and biodistribution with high with an overall uptake of 5.2%ID/g. In general, the tumor-to-organ ratios are high after 1 h p.i. which is shown by, e.g., tumor-to-blood (9.2 ± 1.1), tumor-to-large intestine (24.9 ± 1.7) and tumor-to-muscle (11.5 ± 2.2) ratios (Fig. 8b). Uptake in other organs are also low such as in heart, lungs, liver, spleen, pancreas, stomach, fat and skin. In addition to the tumor accumulation, a slightly higher accumulation at the bones and small intestine were found, which cannot yet be fully explained. One suggestion could be that FAP is also expressed in these tissues. However, it is important that the main accumulation is located in the tumor and although the unexpected radiotracer uptake in bone and small intestine the tumor-to-bone (1.5 ± 0.2) and tumor-to-small intestine (2.9 ± 0.8) ratios are still high enough to provide high contrast PET images.

## Discussion

Two novel bifunctional chelator-linker conjugates based on the FAP inhibitor UAMC1110 were developed. As bifunctional chelators, macrocyclic DOTA as well as the hybrid chelator DATA^5m^ were used. DATA is well known for fast and stable complexation of gallium-68 and to show high stabilities over a long period of time. DOTA is interesting because of its ability to complex other radiometals preferring higher coordination numbers, e.g., the long-lived therapy radionuclide lutetium-177 but also shorter-lived therapeutic radionuclides such as bismuth-213, lead-210 and yttrium-90. DOTA.SA.FAPi allows to use the same precursor for both diagnosis with gallium-68 and therapy with lutetium-177 in nuclear medicine. SA is the main component of the linker system forming a squaramide unit and accordingly substitutes the heterocyclic nitrogen moieties in the structures reported by the Heidelberg group (Lindner et al. 2018; Loktev et al. 2019; Loktev et al. 2018). In recent works from our group, SA has shown good results, both in chemistry and physiologically, as a linker unit coupled with PSMA inhibitors (Greifenstein et al. 2020; Greifenstein et al. 2019). The asymmetrically substituted squaramide unit in the target compounds was efficiently installed with SADE, relying on the latter’s elegant pH-dependent chemistry and selectivity for primary amines. Due to this selectivity, no protecting groups are required. In addition, the pH dependent reactivity of SADE is explained by changes of the aromatic stabilization energy in the ring system over the course of the sequential amidation steps (Tietze et al. 1991). After the first amidation, which is carried out at neutral pH, the obtained uncharged monoamide is characterized by a higher aromatic stabilization than the starting material, squaric acid diester. This stabilization prevents addition of a second amine molecule. By increasing the pH, the monoamide is deprotonated, loses aromatic stabilization, and this allows for addition of a second amine molecule to provide the diamide (Quiñonero et al. 2000; Wurm and Klok 2013). Correspondingly, C_2_-symmetric diamide derivatives of SA can be obtained, if the reaction is performed at higher pH: in that case, two equivalents of amine will directly substitute the ethoxy groups in SADE.

Both DOTA.SA.FAPi and DATA^5m^.SA.FAPi could be well used for radiolabeling with gallium-68. DATA has already demonstrated good complexation with gallium-68 and has the advantage of labeling even under mild conditions such as room temperature. Quantitative gallium-68 labeling results were observed for both FAPi-conjugates with gallium-68. The DOTA complex showed quantitative radiochemical yields at temperatures of 95 °C with precursor amounts of > 15 nmol. For the DATA^5m^ conjugate quantitative yields could be achieved at room temperature for amounts > 15 nmol. In addition, it could be seen that with lower competing cations of gallium-68, less precursor amount (≥ 10 nmol) is required to achieve quantitative complexation, whereas with higher gallium activity (> 200 MBq) more substance > 15 nmol is needed for quantitative yields.

The stability for both derivatives against different media was high with > 95% intact conjugates. [^68^Ga]Ga-DOTA.SA.FAPi proved stable in EtOH, HS and saline and was stable against transmetallation (Cu, Mg, Ca and Fe) and transchelation (DTPA and EDTA). Stability of [^68^Ga]Ga-DATA^5m^.SA.FAPi in HS, PBS and in saline is very high with > 95% intact conjugates over a period of 2 h.

All five measured compounds, DOTA.SA.FAPi, [^nat^Ga]Ga-DOTA.SA.FAPi, [^nat^Lu]Lu-DOTA.SA.FAPi, DATA^5m^.SA.FAPi and [^nat^Ga]Ga-DATA^5m^.SA.FAPi demonstrated very high affinity to FAP with low nanomolar IC_50_ values and high IC_50_ values with respect to PREP. Therefore, all measured FAP inhibitors have a potency in the same order of magnitude as the original FAP inhibitor. In addition, they all presented excellent selectivity for FAP with regard to PREP.

Preclinical in vivo animal studies were performed with HT-29 xenograft mice. The HT-29 cancer cell line is a human colorectal adenocarcinoma cell line with epithelial morphology (Cheng et al. 2002; Henry et al. 2007). When inoculated into nude mice, they produce undifferentiated tumors with modest stroma. Significant FAP expression is present in this stroma, that typically trabeculates between nests of HT-29 cells. Notably, and similar to the situation in most tumor types, FAP staining is distinctly absent from the actual HT-29 cancer cells within the tumors (Cheng et al. 2002).

The highest accumulation in the tumor was found in both the PET images with SUV_mean_ of 0.75 ± 0.09 60 min post injection and biodistribution with an overall uptake of 5.2%ID/g. In addition, the tumor-to-organ ratios in the biodistribution data were quite high (tumor-to-blood (9.2 ± 1.1), tumor-to-large intestine (24.9 ± 1.7) and tumor-to-muscle (11.5 ± 2.2)), which is also reflected by the high contrast in the images. Besides the high tumor uptake, accumulation in the bladder could also be observed, suggesting renal clearance to be the predominant excretion.

The reference FAPI-04 could not be included in the experiments because it was not available yet. While head-to-head comparison with ^68^Ga-FAPI-04 was not possible, we may compare the SUV_mean_ value data from reference publications. It should be noted, however, that different tumor models in different test series were used. Therefore, a direct comparison of the results is not advisable, but tendencies could still be observed. The tumor model used in Heidelberg are HT-1080-FAP cells (transfected fibrosarcoma cells) (Lindner et al. 2018). There is a recent publication from Watabe et al. on ^64^Cu-, ^68^Ga-, and ^225^Ac-FAPi-04 in PANC-1 and MIA PaCa-2 (human pancreatic cancer cells) xenograft tumor mice (Watabe et al. 2020). Table 2 shows a summary of the different models with the corresponding gallium-68 tracer complex and the SUV_mean_ values of PET measurements at 60 min p.i..

**Table 2:** Comparison of [^68^Ga]Ga-FAPI-04 and [^68^Ga]Ga-DOTA.SA.FAPi: SUV_mean_ values of μPET measurements at 60 min p.i. with the corresponding tumor models. The values of the FAPI-04 component are obtained from the reference literatures (Lindner et al. 2018; Watabe et al. 2020)

Compound	[^68^Ga]Ga-FAPI-04		[^68^Ga]Ga-DOTA.SA.FAPi
Tumormodel	HT-1080 FAP	PANC-1 / MIA-PaCa-2	HT-29
**Organ**	**SUV**_**mean**_**(60 min p.i.)**		
Heart	0.16	0.17	0.15
Muscle	0.06	0.03	0.18
Kidney	0.33	0.36	0.27
Liver	0.11	0.67	0.22
Tumor	0.45	0.14 / 0.11	0.75

The values from references were read from the graphs and therefore do not indicate precise values

Table 2 shows that comparable SUV_mean_ values can be found with [^68^Ga]Ga-DOTA.SA.FAPi and [^68^Ga]Ga-FAPI-04 in the HT-1080 FAP model. In both, tumor uptake is highest with overall low background in normal organs. The uptake in the tumor of ^68^Ga-FAPI-04 in the models of Watabe et al. are marginal while liver shows slightly higher uptake.

## Conclusion

In this work, two potential theranostic radiopharmaceuticals were successfully synthesized, based on the selective FAP-inhibitor UAMC1110. Key elements of these compounds are a squaramide motif (introduced via amidation of SADE) and a DOTA or DATA^5m^-type chelator. Due to the unique chemistry of SADE, it was possible to avoid complex synthesis routes and protective group strategies. DOTA.SA.FAPi and DATA^5m^.SA.FAPi showed very good in vitro complexations of gallium-68 and a very high stability in different media of more than 95% intact conjugate. In general, the hybrid chelator DATA^5m^ shows a quantitative complexation under mild conditions and is therefore very well suited to label temperature sensitive target molecules with radiometals.

Both FAPi-precursors as well as their gallium and lutetium versions showed excellent affinity and selectivity to FAP, in the low nanomolar range, with IC_50_-values between 0.7 and 1.4 nM. Conversely, PREP IC_50_-values were found to be in the μM-range, implying excellent FAP/PREP selectivity indices.

In the HT-29 colon cancer xenograft model, first proof-of-concept animal studies with [^68^Ga]Ga-DOTA.SA.FAPi showed good tumoral accumulation with high uptake (SUV_mean_ 0.75 ± 0.09) at 60 min p.i. Ex vivo biodistribution revealed 5.2 ± 0.2% ID/g on average and low background activity, i.e. an overall good tumor-to-organ ratio. Comparison of the different tumor models with the reference compound FAPI-04 has shown that DOTA.SA.FAPi offers comparable results to FAPI-04. The values should not be compared in direct relation, as there are different test series as well as different tumor models, but nevertheless a tendency can be exhibited.

The potential of the novel compound family to target FAP could be clearly demonstrated. The introduction of squaric acid as linker forming a squaramide bond between bifunctional chelator and pharmacophore firstly simplified the preparative work and secondly showed pharmacological improvements due to the excellent in vitro binding affinities and the great in vivo/ ex vivo data. Further preclinical characterizations for both precursors are planned for publication at a later stage. In meantime, a first clinical trial was carried out in cooperation with the University Medical Center Bonn showing specific uptake in focal nodular hyperplasia (Kreppel et al. 2020). Further patient investigations are ongoing and we expect that our FAPi based radiotracers could be of importance characterize various malignant and benign tumor types in nuclear medicine.

## Materials and methods

### Reagents and instrumentations for synthesis

All basic chemicals were acquired from Sigma-Aldrich (St. Louis, USA), Merck KGaA (Darmstadt, Germany), TCI Deutschland GmbH (Eschborn, Germany) and VWR International GmbH (Darmstadt, Germany). DOT3AtBu-*N*-(2-aminoethyl) ethanamide **1** was purchased from CheMatech (Dijon, France), (*S*)-6-(4-aminobutoxy)-*N*-(2-(2-cyano-4,4-difluoropyrrolidin-1-yl)-2-oxoethyl)-quinoline-4-carboxamide **3** was purchased from KE Biochem Co. (Shanghai, China). Thin-layer chromatography plates from Merck, Kieselgel 60 F254 coated aluminum plates, were used for the analysis. Detection was carried out by fluorescence extinction at λ = 254 nm and by staining with potassium permanganate. Silica gel 60 (core size 0.063 0.200 mm) from Acros Organics (Schwerte, Germany) was used for purification by column chromatography. The LC/MS spectra were measured on an Agilent Technologies 1220 Infinity LC system coupled to an Agilent Technologies 6130B Single Quadrupole LC/MS system. The ^1^H and ^13^C NMR measurements were performed at 400 MHz (400 MHz FT NMR spectrometer AC 400, Bruker Analytik GmbH). For analytical and semi-preparative HPLC a 7000 series Hitachi LaChrom with a Phenomenex (Aschaffenburg, Germany) Luna C18 (250 × 4.6 mm, 5 μ) column, a Phenomenex Luna C18 (250 × 10 mm, 10 μ) column and a Phenomenex Synergi C18 (250 × 10 mm, 4 μ) column were used.

### Organic synthesis

#### Synthesis of DOTA.SA.FAPi

##### DOTA.SA (**2**) [2,2′,2″-(10-(2-((2-((2-ethoxy-3,4-dioxocyclobut-1-en-1-yl)amino)ethyl)amino)-2-oxoethyl)-1,4,7,10-tetraazacyclododecane-1,4,7-triyl) triacetic acid]

**1** (48.0 mg; 78.1 μmol) was reacted with 1 mL 80% TFA in DCM for 6 h at room temperature for deprotection of *tert*-butyl protecting groups. After evaporating TFA/DCM, the residue was reacted with 3,4-diethoxycyclobut-3-ene-1,2-dione (13.3 mg; 78.1 μmol) in 500 μL 0.5 M Na_2_HPO_4_/NaH_2_PO_4_ phosphate buffer pH 7 and shaken at room temperature overnight. The chelator-linker conjugate DOTA.SA **2** could be isolated via HPLC purification. After HPLC purification (Phenomenex® Luna® 10 μm C18 (2) 100 Å, gradient 6–8% MeCN (+ 0.1% TFA)/ 94–92% water (+ 0.1% TFA) in 20 min with a 5 mL/min flow) and lyophilization the product was obtained as white powder (28.2 mg; 49.4 μmol; 63%). ^1^H-NMR (D_2_O, 600 MHz, δ [ppm]): 4.64–4.53 (dq, 2 H); 3.93–2.89 (m, 28 H); 1.41–1.33 (m, 3 H). MS (ESI^+^): m/z (%): 571.3 (M + H^+^), 593.3 (M + Na); calculated for C_24_H_38_N_6_O_10_: 570.26

##### DOTA.SA.FAPi (**4**) [(S)-2,2′,2″-(10-(2-((2-((2-((4-((4-((2-(2-cyano-4,4-difluoropyrrolidin-1-yl)-2-oxo-ethyl)carbamoyl)quinolin-6-yl)oxy)butyl)amino)-3,4-dioxocyclobut-1-en-1-yl)amino)ethyl)amino)-2-oxoethyl)-1,4,7,10-tetraazacyclododecane-1,4,7-triyl) triacetic acid]

Coupling of DOTA.SA **2** (10.3 mg; 17.5 μmol) and NH_2_-UAMC1110 **3** (11.4 mg; 26.3 μmol) to form DOTA.SA.FAPi **4** was performed by amidation at pH 9 in 500 μL 0.5 M Na_2_HPO_4_ phosphate buffer at room temperature. The reaction was shaken for 12 h. DOTA.SA.FAPi **4** was isolated via HPLC purification (Phenomenex® Luna® 10 μm C18(2) 100 Å) with a linear gradient condition of 15–20% MeCN (+ 0,1% TFA)/85–80% Water (+ 0,1% TFA) in 20 min with a 5 mL/min flow. After lyophilization the product was obtained as yellow powder (12.2 mg; 12.7 μmol, 73%). MS (ESI^+^): m/z (%): 956.4 (M + H^+^), 978.4 (M + Na); calculated for C_43_H_55_F_2_N_11_O_12_: 955.40.

#### Synthesis of DATA^5m^.SA.FAPi

##### DATA^5m^-en [1,4-Di (tert-butylacetate)-6-((5-(2-((tert-butoxy-carbonyl)aminoethyl)amino)-5-oxopentyl)-6-(amino (methyl)-tert-butylacetate)-perhydro-1,4-diazepane] (**6**)

**5** (100 mg; 0.18 mmol) was added to 1 mL dry MeCN, HATU (66.3 mg; 0.18 mmol), HOBt (70.9 mg; 0.53 mmol) and DIPEA (89.3 μL; 0.53 mmol) were added and stirred for 1 h at room temperature. *N*-boc-ethylenediamine (56.1 mg; 0.35 mmol) was added to the solution and stirred overnight. After completion of the reaction, the solution was concentrated under vacuum and the residue was purified by column chromatography (CHCl_3_/MeOH, 20:1, Rf = 0.23). The product was obtained as yellow oil (114 mg; 0.16 mmol; 91%). ^1^H-NMR (DMSO, 400 MHz, δ [ppm]): 3.36 (s, 2 H); 3.23 (s, 4 H); 3.07–3.01 (m, 2 H); 2.97–2.91 (m, 2 H); 2.79 (d, J = 13.7 Hz, 2 H); 2.72–2.67 (m, 2 H); 2.59–2.54 (m, 2 H); 2.51 (d, J = 13.7 Hz, 2 H); 2.17 (s, 3 H); 2.03 (t, 2 H); 1.45–1.41 (m, 4 H); 1.40 (s, 18 H); 1.39 (s, 9 H); 1.37(s, 9 H); 1.22–1.18 (m, 2 H). ^13^C-NMR (CDCl_3_, 100 MHz, δ [ppm]): 172.25 (s); 171.72 (s); 170.28 (s); 169.58 (s); 155.62 (s); 80.19 (s); 80.08 (s); 77.63 (s); 62.37 (s); 61.87 (s); 61.73 (s); 58.72 (s); 56.06 (s); 51.50 (s); 37.10 (s); 35.55 (s); 28.24 (s); 27.87 (s); 27.77 (s); 26.11 (s); 25.50 (s); 21.55 (s). MS (ESI^+^): m/z (%): 714.4 (M + H^+^); 736.5 (M + Na^+^); calculated for C_36_H_67_N_5_O_9_: 713.49

##### DATA^5m^.SA [1,4-Di (acetate)-6-((5-(2-((2-ethoxy-3,4-dioxo-cyclobut-1-en-1yl)aminoethyl)amino)-5-oxo-pentyl)-6-(amino (methyl)-acetate)-perhydro-1,4-diazepane] (**7**)

**6** (100 mg; 0.14 mmol) was dissolved in DCM/TFA (1:1; vol%) and stirred for 3 h. after complete deprotection of the *tert*-butyl groups, the solution was concentrated under vacuum and 3 mL 0.5 M phosphate buffer pH 7 was added to the residue. After adding 3,4-diethoxycyclobut-3-ene-1,2-dione (61.7 μL; 0.42 mmol) to the solution, the pH was adjusted again to pH 7 with 1 M NaOH and stirred overnight at room temperature. After completion, the reaction solution was purified by HPLC (Phenomenex® Luna® 10 μm C18(2) 100 Å) with a linear gradient condition of 8–12% MeCN (+ 0,1% TFA)/92–88% water (+ 0,1% TFA) in 20 min with a 5 mL/min flow. After lyophilization the product was obtained as white powder (24.8 mg; 43.6 μmol, 31%). ^1^H-NMR (D_2_O, 600 MHz, δ [ppm]): 4.73–4.66 (m, 2 H); 3.79 (s, 2 H); 3.70 (s, 4 H), 3.67–3.47 (m, 6 H); 3.39–3.22 (m, 6 H); 2.98 (d, J = 8.7 Hz, 3 H); 2.22 (t, 2 H); 1.71–1.68 (m, 2 H); 1.53–1.48 (m, 2 H); 1.43–1.38 (m, 2 H); 1.35–1.29 (m, 2 H). ^13^C-NMR (D_2_O, 150 MHz, δ [ppm]): 188.70 (s); 183.25 (s); 177.21 (s); 176.42 (s); 173.82 (s); 170.00 (s); 117.19 (s); 115.26 (s); 70.66 (s); 68.77 (s); 54.14 (s); 43.89 (s); 39.22 (s); 37.76 (s); 35.09 (s); 29.53 (s); 25.69 (s); 25.54 (s); 22.09 (s); 15.03 (s); 14.94 (s). MS (ESI^+^): m/z (%): 570.3 (M + H^+^); 593.3 (M + Na^+^); calculated for C_25_H_39_N_5_O_10_: 569.27

##### DATA^5m^.SA.FAPi (**8**) [(S)-2,2′-(6-((carboxymethyl)(methyl)amino)-6-(5-((2-((2-((4-((4-((2-(2-cyano-4,4-difluoropyrrolidin-1-yl)-2-oxoethyl)carbamoyl)quinolin-6-yl)oxy)butyl)amino)-3,4-dioxocyclobut-1-en-1-yl)amino)ethyl)amino)-5-oxopentyl)-1,4-diazepane-1,4-diyl) diacetic acid]

DATA^5m^.SA **7** (8.7 mg, 15.3 μmol) and NH_2_-UAMC1110 **3** (19.8 mg, 45.9 μmol) were reacted to form DATA^5m^.SA.FAPi **8** via amidation at pH 9 in 500 μL 0.5 M Na_2_HPO_4_ phosphate buffer at room temperature stirred overnight. DATA^5m^.SA.FAPi was isolated via HPLC purification (Phenomenex® Luna® 10 μm C18(2) 100 Å) with a linear gradient condition of 18–20% MeCN (+ 0,1% TFA)/82–80% Water (+ 0,1% TFA) in 20 min. The product was obtained as yellowish powder (6.2 mg, 6.5 μmol; 42%). MS (ESI^+^): m/z (%): 955.4 (M + H^+^); calculated for C_44_H_56_F_2_N_10_O_12_: 954.40.

### ^nat^Ga/ ^nat^Lu-complexes of DOTA.SA.FAPi

The ^nat^Ga-metallated species [^nat^Ga]Ga-DOTA.SA.FAPi was obtained after treatment of DOTA.SA.FAPi (5.2 mg; 5.4 μmol) with stoichiometric amount (1 eq) of 10 mM ^nat^Ga (NO_3_)_2_ in 1 mL 0.2 M AmAc buffer pH 4.5 shaken for 3 h at 80 °C. Complexation was confirmed by ESI-MS and HPLC-purification was performed (Phenomenex® Synergi® 10 μm (C18) 100 Å (250 mm × 10 mm, 10 μm), linear gradient of 5–95% MeCN (+ 0,1% TFA)/95–5% Water (+ 0,1% TFA) in 10 min. The product was obtained as yellowish powder (4.6 mg, 4.5 μmol; 83%). MS (ESI^+^): m/z (%): 1022.2 (M + H^+^), 1044.2 (M + Na); calculated for C_43_H_53_F_2_GaN_11_O_12_: 1021.30.

The ^nat^Lu-metallated species [^nat^Lu]Lu-DOTA.SA.FAPi was obtained after treatment of DOTA.SA.FAPi (6.0 mg; 6.3 μmol) with stoichiometric amount (1 eq) of 1 mM ^nat^LuCl_3_ in 1 mL 0.2 M AmAc buffer pH 4.5 shaken for 3 h at 80 °C. Complexation was confirmed by ESI-MS and HPLC purification was done analogously to the gallium species. The product was obtained as yellowish powder (5.5 mg, 4.9 μmol; 77%). MS (ESI^+^): m/z (%): 1028.3 (M + H^+^), 1051.2 (M + Na); calculated for C_43_H_52_F_2_LuN_11_O_12_: 1027.32.

### ^nat^Ga-complexes of DATA^5m^.SA.FAPi

The ^nat^Ga-metallated species [^nat^Ga]Ga-DATA^5m^.SA.FAPi was obtained after treatment of DATA^5m^.SA.FAPi (7.2 mg; 7.5 μmol) with stoichiometric amount of ^nat^Ga (NO_3_)_2_ in 1 mL 0.2 M AmAc buffer pH 4.5 shaken for 2 h at 25 °C. Complexation was confirmed by ESI-MS and HPLC-purification was performed (Phenomenex® Luna® 10 μm (C18) 100 Å (250 mm × 10 mm, 10 μm), linear gradient of 5–95% MeCN (+ 0,1% TFA)/95–5% Water (+ 0,1% TFA) in 10 min. The product was obtained as yellowish powder (4.4 mg, 4.3 μmol; 57%). MS (ESI^+^): m/z (%): 1021.3 (M + H^+^), 1043.2 (M + Na); calculated for C_44_H_53_F_2_GaN_10_O_12_: 1020.31.

### Inhibitory potency determination

Enzymes**:** A gateway-entry clone for human FAP was purchased from Dharmacon (Accession number DQ891423) and the human secretion signal was replaced with the HoneyBee mellitin secretion signal. For transfection and expression of FAP in Sf9 insect cells, the C-terminal BaculoDirect kit from LifeTechnologies was used. The enzyme was purified from the supernatant of the insect cells using immobilized Ni-chelating chromatography (GE healthcare, Diegem, Belgium), followed by anion-exchange chromatography using a 1 mL HiTrap Q (GE healthcare, Diegem, Belgium). Human recombinant PREP was expressed in BL21(DE3) cells and purified using immobilized Co-chelating chromatography (GE healthcare) followed by anion-exchange chromatography on a 1 ml Mono Q column (GE healthcare).

FAP: IC_50_ measurements of the inhibitors were carried out using Z-Gly-Pro-7-amino-4-methylcoumarine (AMC) (Bachem, Switzerland) as the substrate at a concentration of 50 μM at pH 8 (0.05 M Tris-HCl buffer with 0.1% glycerol, 1 mg/mL BSA and 140 mM NaCl). Eight concentrations of inhibitors were tested. The final DMSO concentration was kept constant during the experiment to exclude any solvent effects. Inhibitors were pre-incubated with the enzyme for 15 min at 37 °C, afterwards the substrate was added and the velocities of AMC release were measured kinetically at λ_ex_ = 380 nm, λ_em_ = 465 nm for at least 10 min at 37 °C. The Infinite 200 (Tecan Group Ltd.) micro-titer plate reader and the Magellan software were used for measurement and data processing respectively.

Note: a slightly different protocol, involving a different FAP substrate (Ala-Pro-pNA), was used to determine the originally published FAP IC_50_-value for reference UAMC1110 (3.2 +/− 0.4 nM). This accounts for the non-identical value published here.

PREP: IC_50_ measurements of the inhibitors were carried out using *N*-succinyl-Gly-Pro-AMC (Bachem, Switzerland) as the substrate at a concentration of 250 μM at pH 7.4 (0.1 M K-phosphate, 1 mM EDTA, 1 mM DTT). Eight concentrations of inhibitors were tested. The final DMSO concentration is kept constant during the experiment to exclude any effects. Inhibitors were pre-incubated with the enzyme for 15 min at 37 °C, afterwards the substrate was added and the velocities of AMC release were measured kinetically at λ_ex_ = 380 nm, λ_em_ = 465 nm for at least 10 min at 37 °C. The Infinite 200 (Tecan Group Ltd.) micro-titer plate reader and the Magellan software were used for measurement and data processing, respectively.

The data were fitted using a non-linear fit model in GraFit 7 software, according to the following equation:

$$ y=\frac{range}{1+{\left(\frac{x}{IC_{50}}\right)}^s} $$

where y is the value of the residual enzymatic activity compared to a non-inhibited sample, x is the final inhibitor concentration in the assay, s is the slope factor and the IC_50_ is the half maximal inhibitory concentration.

### Radiolabeling and stability studies with gallium-68

Gallium-68 was obtained manually utilizing ethanol-based post-processing from a ^68^Ge/^68^Ga-generator (ITG Garching, Germany). Elution process was performed following the protocol established by Eppard et al. (Eppard et al. 2014). After elution of gallium through the generator with a 0.05 M HCl (5 mL) solution gallium-68 was distributed on the microchromatography CEX column AG 50 W-X4. The column was washed with 1 mL 80% EtOH/ 0.15 M HCl and the Ga (III) was eluted from the column with 400 μL 90% EtOH/ 0.9 M HCl. The washing step ensures that unwanted chemical and radiochemical impurities are separated and only ^68^Ga^3+^ remains on the column.

Reaction controls for radiochemical purity were executed using radio-TLC (TLC Silica gel 60 F_254_ Merck) with citrate buffer pH 4 and radio-HPLC using an analytical HPLC 7000 series Hitachi LaChrom with a Phenomenex (Aschaffenburg, Germany) Luna C18 column (250 × 4.6 mm, 5 μ), linear gradient of 5–95% MeCN (+ 0,1% TFA)/ 95–5% Water (+ 0,1% TFA) in 10 min). TLC’s were measured in TLC imager CR-35 Bio Test-Imager from Duerr-ndt (Bietigheim-Bissingen, Germany) with the analysis software AIDA Elysia-Raytest (Straubenhardt, Germany). The citrate TLCs show free radio metal with a R_f_ value of 0.8–0.9. The labeled complexes are observed at a R_f_ value of 0.1–0.2.

Gallium-68 stability studies against transmetallation (Fe, Cu, Ca, Mg), transchelation (EDTA, DTPA) as well as in HS, EtOH and saline (0.9% isotone NaCl-solution) were performed. 50 μl of the [^68^Ga]Ga-DOTA.SA.FAPi labeling solution with > 95% radiochemical purity were added to 1 mL of the respective media. The measured time points for gallium-68 were 15, 30, 45, 60, 90, 120 min. HS (human male AB plasma, USA origin) was bought from Sigma Aldrich, PBS was purchased from Sigma Aldrich and 0.9% saline from B. Braun Melsungen AG (Melsungen, Germany).

### In vivo animal studies and ex vivo biodistributions

After quantitative radiolabeling of [^68^Ga]Ga-DOTA.SA.FAPi with a tracer amount of 20 nmol at 95 °C in 20 min. The solution was purified via C-18 column (Sep-Pak Light C18, Waters Corporation, Massachusetts, USA). Conditioning of the SPE was performed using 5 ml abs. Ethanol and 5 mL water. Crude reaction mixture was pressed over the SPE and then washed with 5 ml water. Afterwards, the gallium-68 labeled product was eluted with 1 mL of 50 vol% ethanol. Finally, the ethanol was evaporated and the tracer was reformulated in 5% ethanol in saline solution (500 μl total volume). The radiochemical purity was > 99% and no traces of free gallium could be detected by radio-TLC analysis (mobile phase: citrate buffer pH 4.0) and RP-HPLC (5–95% MeCN (+ 0,1% TFA)/95–5% Water (+ 0,1% TFA) in 10 min). The activity after purification process was 200 ± 10 MBq (10 GBq/ μmol) with a total RCY of 56%.

In vivo tumor model: HT-29 (human colon adenocarcinoma, ATCC, Rockville, Maryland) cells were routinely cultured in Dulbecco’s Modified Eagle Medium supplemented with 10% heat inactivated foetal bovine serum (FBS), 2 mM glutamine, 1% sodium pyruvate and 1% penicillin/streptomycin (Gibco, Life technologies). After detaching the cells, the number of viable cells was counted with the automated Muse™ Cell Analyzer (Merck Millipore). For the HT-29 subcutaneous model, 10.10^6^ viable cells, suspended in 100 μl PBS, were inoculated in the right hind leg of female 6-week-old CD1^−/−^ Foxn1nu mice (*n* = 3), obtained from Charles River Laboratories (L’Arbresle, France). The animals were kept under environmentally controlled conditions (12 h light/dark cycle, 20–24 °C and 40–70% relative humidity) with food and water ad libitum. When tumors reached an approximate volume of 400 mm^3^, 3 mice underwent μPET imaging. All experimental procedures and protocols were performed in accordance with European Directive 86/609/EEC Welfare and Treatment of Animals and were approved by the local ethical commission (2017–070, University of Antwerp, Belgium).

Micro-PET imaging: Micro-PET scans were carried out using an Inveon small-animal PET/CT scanner (Siemens), after i.v. injection of 4 nmol of [^68^Ga]-DOTA.SA.FAPi (8.6 MBq, molar activity of 2.1 GBq/ μmol) into tumor bearing mice (*n* = 3), under isoflurane anesthesia (5% for induction, 2% for maintenance). Static whole-body PET images were acquired 60 min after injection of the radiotracer. Following each PET acquisition, a whole-body CT scan was acquired to obtain the animal’s anatomical information individually.

For quantitative analysis, PET data were reconstructed using 3-dimensional ordered subset expectation maximization (OSEM3D, 16 subsets and 2 iterations) and 18 maximum a posteriori (MAP) iterations including scatter and attenuation correction (matrix size, 128 × 128 × 159; voxel size, 0.776 × 0.776 × 0.776 mm;). Volumes of interest (VOIs) were manually drawn on the PET/CT images using PMOD (version 3.6; PMOD Technologies) to delineate the tumor, heart and muscle.

Ex vivo biodistribution: Immediately after the CT scans, the animals were sacrificed, the blood, tissues and organs were collected, weighed and the radioactivity was measured using an automatic γ-counter (Wizard^2^ 2480, PerkinElmer). Values were expressed as percentage of the injected dose per gram (%ID/g).

## Supplementary information

**Additional file 1: Figure S1.** HPLC spectra of DOTA.SA.FAPi with linear gradient condition of 5–95% MeCN (+ 0.1% TFA)/95–5% Water (+ 0.1% TFA) in 10 min, 1 mL/min, t_R_ = 8.6 min. **Figure S2.** HPLC spectra of DATA^5m^.SA.FAPi with linear gradient condition of 5–95% MeCN (+ 0.1% TFA)/95–5% Water (+ 0.1% TFA) in 10 min, 1 mL/min, t_R_ = 8.5 min. **Figure S3.** radiolabeling kinetics at different temperatures of [^68^Ga]Ga-DOTA.SA.FAPi complex. **Figure S4.** Stability studies for [^68^Ga]Ga-DOTA.SA.FAPi complex in human serum, Ethanol and 0.9% isotone NaCl-solution in % of intact conjugate at different time points. **Figure S5.** Stability studies for [^68^Ga]Ga-DOTA.SA.FAPi complex against transmetallation (Fe, Cu, Mg and Ca) in % of intact conjugate at different time points. **Figure S6.** Stability studies for [^68^Ga]Ga-DOTA.SA.FAPi complex against transchelation (DTPA and EDTA) in % of intact conjugate at different time points. **Figure S7.** Stability studies for [^68^Ga]Ga-DATA^5m^.SA.FAPi complex in human serum, Ethanol and 0.9% isotone NaCl-solution in % of intact conjugate at different time points. **Figure S8.** radio-HPLC spectra of DOTA.SA.FAPi with linear gradient condition of 5–95% MeCN (+ 0.1% TFA)/95–5% Water (+ 0.1% TFA) in 8 min, 1 mL/min, t_R_ = 9.1 min. **Figure S9.** Inhibition assay graph and calculated IC_50_-data for DOTA.SA.FAPi (*n* = 3) with regard to FAP. **Figure S10.** Inhibition assay graph and calculated IC_50_-data for ^nat^Ga-DOTA.SA.FAPi (*n* = 3) with regard to FAP. **Figure S11.** Inhibition assay graph and calculated IC_50_-data for ^nat^Lu-DOTA.SA.FAPi (*n* = 3) with regard to FAP. **Figure S12.** Inhibition assay graph and calculated IC_50_-data for DOTA.SA.FAPi (*n* = 3) with regard to PREP. **Figure S13.** Inhibition assay graph and calculated IC_50_-data for ^nat^Ga-DOTA.SA.FAPi (*n* = 3) with regard to PREP. **Figure S14.** Inhibition assay graph and calculated IC_50_-data for ^nat^Lu-DOTA.SA.FAPi (n = 3) with regard to PREP. **Figure S15.** Inhibition assay graph and calculated IC_50_-data for DATA^5m^.SA.FAPi (*n* = 3) with regard to FAP. **Figure S16.** Inhibition assay graph and calculated IC_50_-data for ^nat^Ga- DATA^5m^.SA.FAPi (*n* = 3) with regard to FAP. **Figure S17.** Inhibition assay graph and calculated IC_50_-data for DATA^5m^.SA.FAPi (*n* = 3) with regard to PREP. **Figure S18.** Inhibition assay graph and calculated IC_50_-data for ^nat^Ga-DATA^5m^.SA.FAPi (*n* = 3) with regard to PREP. **Table S1.** Ex vivo biodistribuion data of [^68^Ga]Ga-DOTA.SA.FAPi at 1 h p.i. (*N* = 3).

## Data Availability

Data sharing is not applicable to this article as no datasets were generated. Please contact authors for data request.

## Abbrevations

DATA: 2,2′-(6-((carboxymethyl)amino)-1,4-diazepane-1,4-diyl) diacetic acid).
DATA^5m^-3^t^Bu: 5-[1,4-bis tertbutoxycarbonylmethyl-6-(tert-butoxycarbonylmethyl-methyl-amino)-[1,4] diazepan-6-yl]-pentanoic acid
DOTA: 1,4,7,10-Tetraazacyclododecane-1,4,7,10-tetraacetic acid
DTPA: Cyclohexyldiethylene-triaminepentaacetic acid
EDTA: Ethylenediaminetetraacetic acid
FAP: Fibroblast activation protein
PREP: Prolyl endopeptidase
DPP: Dipeptidyl peptidase
CAF: Cancer associated fibroblast
IC_50_: Half-maximal inhibitory concentration
MIP: Maximum intensity projection
PET: Positron emission tomography
CT: Computed tomography
Boc: *Tert*-Butyloxycarbonyl
^*t*^Bu: *Tert*-Butyl
ESI: Electrospray ionization
HS: Human serum
HPLC: High-performance liquid chromatography
LC: Liquid chromatography
MS: Mass spectrometry
AmAc: Ammonium acetate
NaAc: Natrium acetate
NMR: Nuclear magnetic resonance
PBS: Phosphate buffered saline
RCY: Radiochemical yield
RT: Room temperature
SA: Squaric acid
SADE: Squaric acid diethyl ester
TLC: Thin layer chromatography
AMC: Z-Gly-Pro-7-amino-4-methylcoumarine
FBS: Foetal bovine serum
p.i.: Post injection
