# Supplementary material for: Targeting fibroblast activation protein (FAP): next generation PET radiotracers using squaramide coupled bifunctional DOTA and DATA5m chelators
Source: EJNMMI Radiopharm Chem. 2020 Jul 29;5:19. doi: 10.1186/s41181-020-00102-z (PMC7391456; doi:10.1186/s41181-020-00102-z)
Supplement: Supplementary file 1 — Additional file 1: Figure S1. HPLC spectra of DOTA.SA.FAPi with linear gradient condition of 5–95% MeCN (+ 0.1% TFA)/95–5% Water (+ 0.1% TFA) in 10 min, 1 mL/min, tR = 8.6 min. Figure S2. HPLC spectra of DATA5m.SA.FAPi with linear gradient condition of 5–95% MeCN (+ 0.1% TFA)/95–5% Water (+ 0.1% TFA) in 10 min, 1 mL/min, tR = 8.5 min. Figure S3. radiolabeling kinetics at different temperatures of [68Ga]Ga-DOTA.SA.FAPi complex. Figure S4. Stability studies for [68Ga]Ga-DOTA.SA.FAPi complex in human serum, Ethanol and 0.9% isotone NaCl-solution in % of intact conjugate at different time points. Figure S5. Stability studies for [68Ga]Ga-DOTA.SA.FAPi complex against transmetallation (Fe, Cu, Mg and Ca) in % of intact conjugate at different time points. Figure S6. Stability studies for [68Ga]Ga-DOTA.SA.FAPi complex against transchelation (DTPA and EDTA) in % of intact conjugate at different time points. Figure S7. Stability studies for [68Ga]Ga-DATA5m.SA.FAPi complex in human serum, Ethanol and 0.9% isotone NaCl-solution in % of intact conjugate at different time points. Figure S8. radio-HPLC spectra of DOTA.SA.FAPi with linear gradient condition of 5–95% MeCN (+ 0.1% TFA)/95–5% Water (+ 0.1% TFA) in 8 min, 1 mL/min, tR = 9.1 min. Figure S9. Inhibition assay graph and calculated IC50-data for DOTA.SA.FAPi (n = 3) with regard to FAP. Figure S10. Inhibition assay graph and calculated IC50-data for natGa-DOTA.SA.FAPi (n = 3) with regard to FAP. Figure S11. Inhibition assay graph and calculated IC50-data for natLu-DOTA.SA.FAPi (n = 3) with regard to FAP. Figure S12. Inhibition assay graph and calculated IC50-data for DOTA.SA.FAPi (n = 3) with regard to PREP. Figure S13. Inhibition assay graph and calculated IC50-data for natGa-DOTA.SA.FAPi (n = 3) with regard to PREP. Figure S14. Inhibition assay graph and calculated IC50-data for natLu-DOTA.SA.FAPi (n = 3) with regard to PREP. Figure S15. Inhibition assay graph and calculated IC50-data for DATA5m.SA.FAPi (n = 3) with regard [file 41181_2020_102_MOESM1_ESM.docx]

**SUPPORTING INFORMATION**

**Targeting fibroblast activation protein (FAP):**

**Next generation PET radiotracers using squaramide**

**coupled bifunctional DOTA and DATA^5m^ chelators**

Euy Sung Moon^1^, Filipe Elvas^2^, Gwendolyn Vliegen^3^, Stef De Lombaerde^2^, Christel Vangestel^2^, Sven De Bruycker^5^, An Bracke^3^, Elisabeth Eppard^4^, Lukas Greifenstein^1^, Benedikt Klasen^1^, Vasko Kramer^4^, Steven Staelens^5^, Ingrid De Meester^3^*, Pieter Van der Veken^3^*, Frank Roesch^1^*

^1^Department of Chemistry – TRIGA site, Johannes Gutenberg University Mainz, 55128 Mainz, Germany

^2^Department of Nuclear Medicine, Antwerp University Hospital (UZA), 2650 Edegem, Belgium

^3^Department of Pharmaceutical Sciences, Laboratory of Medical Biochemistry, University of Antwerp, 2610 Wilrijk, Belgium

^4^Positronpharma SA, 7500921 Providencia, Santiago, Chile

^5^Molecular Imaging Center Antwerp (MICA), University of Antwerp, 2610 Wilrijk, Belgium

**Synthesis**


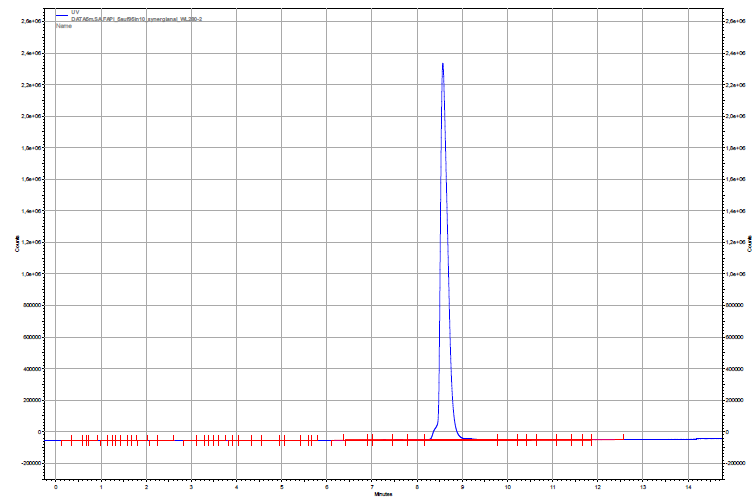


Figure S1: HPLC spectra of DOTA.SA.FAPi with linear gradient condition of 5-95 % MeCN (+0.1 % TFA)/95-5 % Water (+0.1 % TFA) in 10 min, 1 mL/min, t_R_ = 8.6 min.


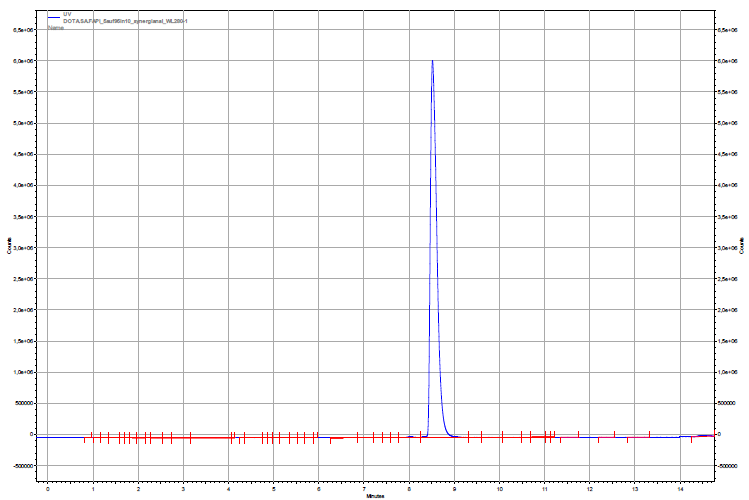


Figure S2: HPLC spectra of DATA^5m^.SA.FAPi with linear gradient condition of 5-95 % MeCN (+0.1 % TFA)/95-5 % Water (+0.1 % TFA) in 10 min, 1 mL/min, t_R_ = 8.5 min.

**Radiolabeling**

Figure S3: radiolabeling kinetics at different temperatures of [^68^Ga]Ga-DOTA.SA.FAPi complex

Figure S4: Stability studies for [^68^Ga]Ga-DOTA.SA.FAPi complex in human serum, Ethanol and 0.9 % isotone NaCl-solution in % of intact conjugate at different time points

Figure S5: Stability studies for [^68^Ga]Ga-DOTA.SA.FAPi complex against transmetallation (Fe, Cu, Mg and Ca) in % of intact conjugate at different time points

Figure S6: Stability studies for [^68^Ga]Ga-DOTA.SA.FAPi complex against transchelation (DTPA and EDTA) in % of intact conjugate at different time points

Figure S7: Stability studies for [^68^Ga]Ga-DATA^5m^.SA.FAPi complex in HS, PBS and 0.9 % isotone NaCl-solution in % of intact conjugate at different time points


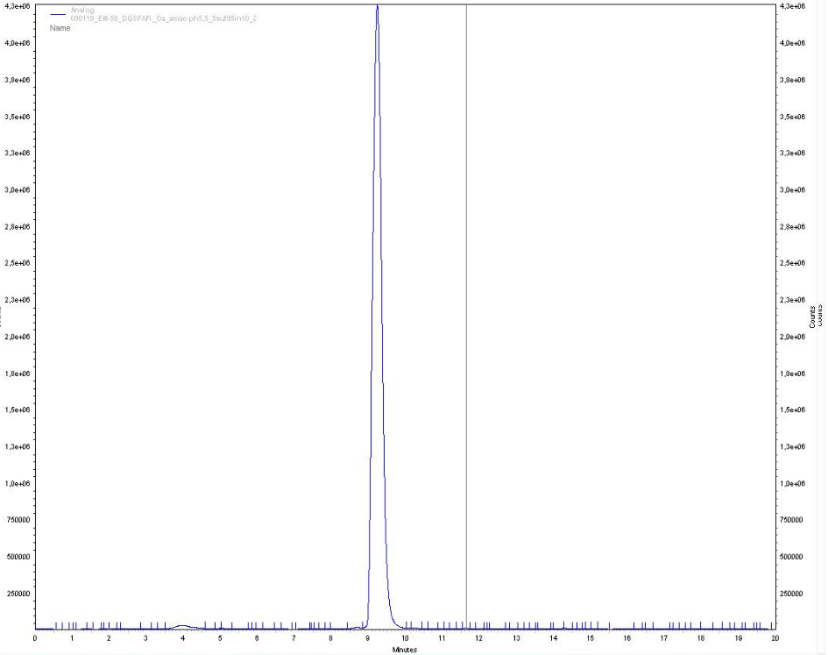


Figure S8: radio-HPLC spectra of DOTA.SA.FAPi with linear gradient condition of 5-95 % MeCN (+0.1 % TFA)/95-5 % Water (+0.1 % TFA) in 8 min, 1 mL/min, t_R_ = 9.1 min.

**Inhibition assays**

*
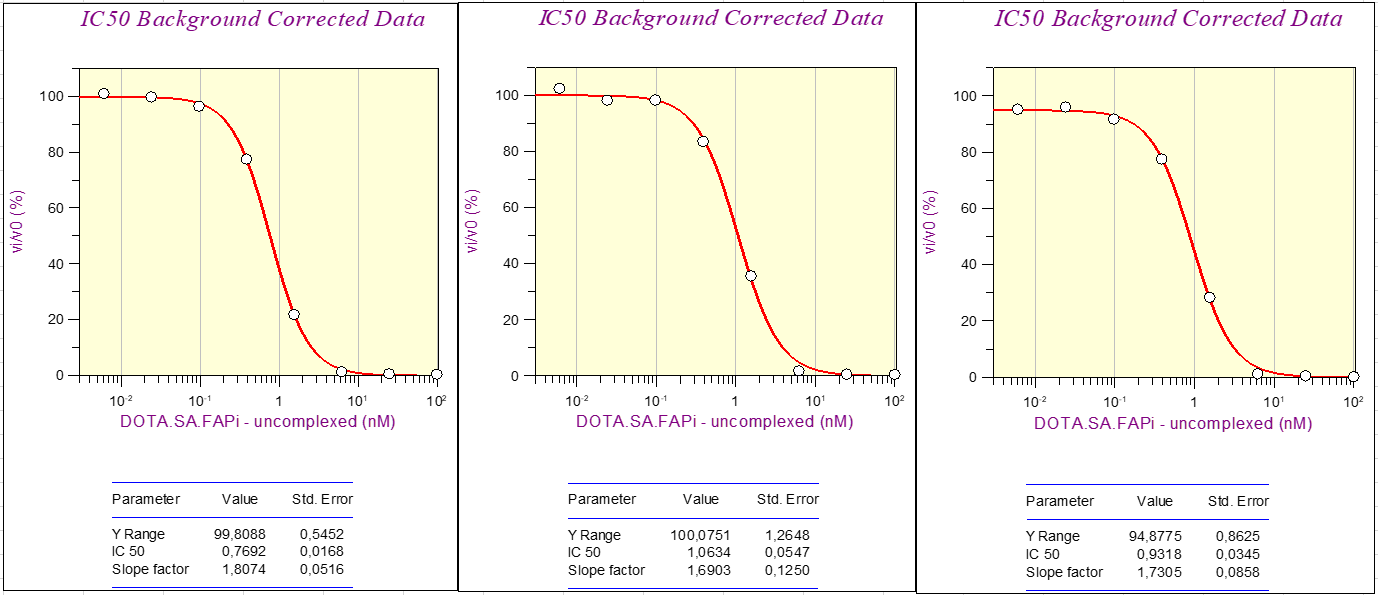
*

Figure S9: Inhibition assay graph and calculated IC_50_-data for DOTA.SA.FAPi (n=3) with regard to FAP.

***
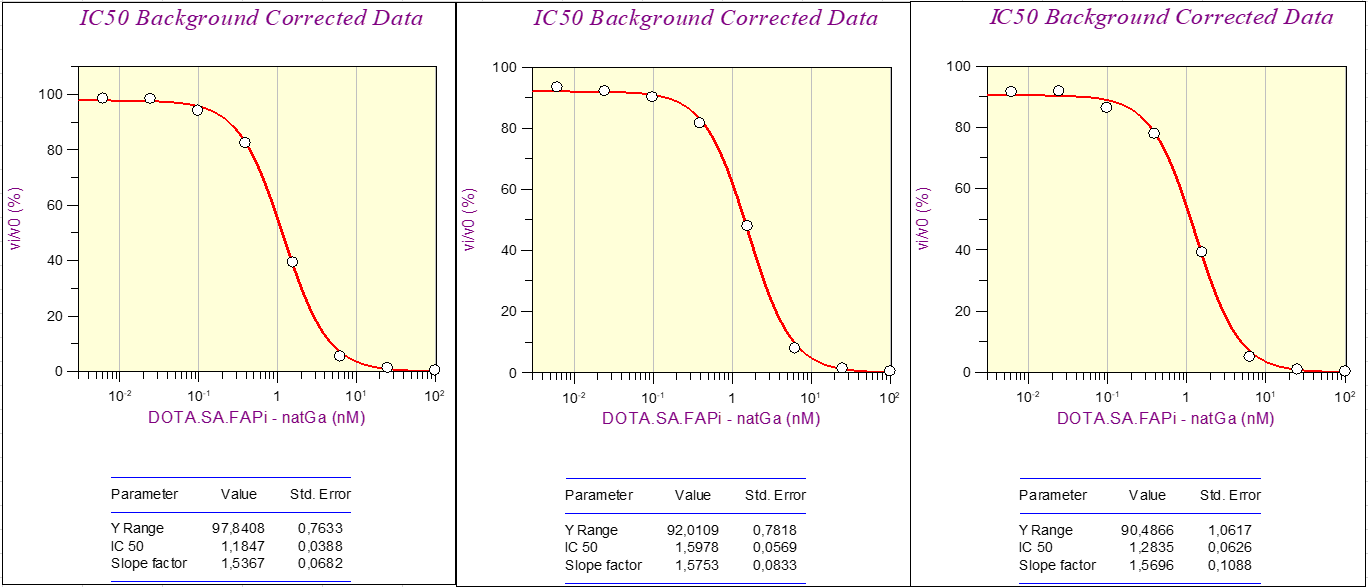
***

Figure S10: Inhibition assay graph and calculated IC_50_-data for ^nat^Ga-DOTA.SA.FAPi (n=3) with regard to FAP.

***
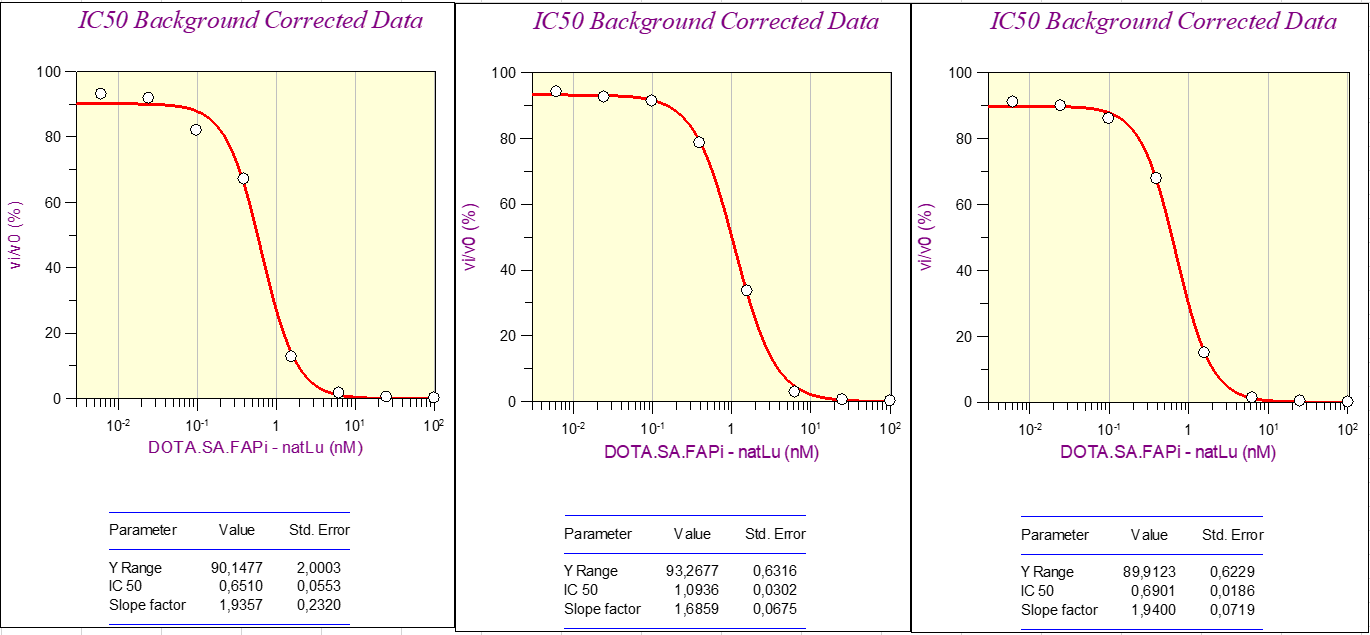
***

Figure S11: Inhibition assay graph and calculated IC_50_-data for ^nat^Lu-DOTA.SA.FAPi (n=3) with regard to FAP.


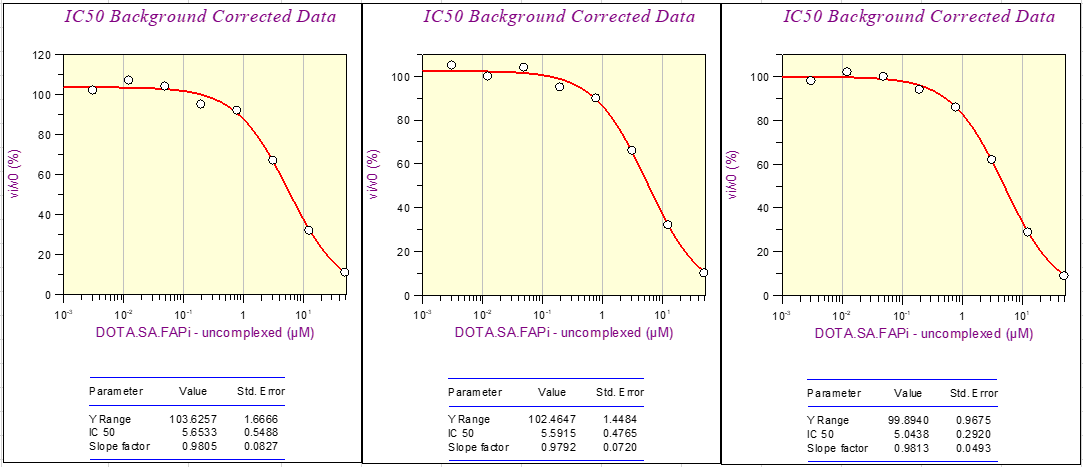


Figure S12: Inhibition assay graph and calculated IC_50_-data for DOTA.SA.FAPi (n=3) with regard to PREP.


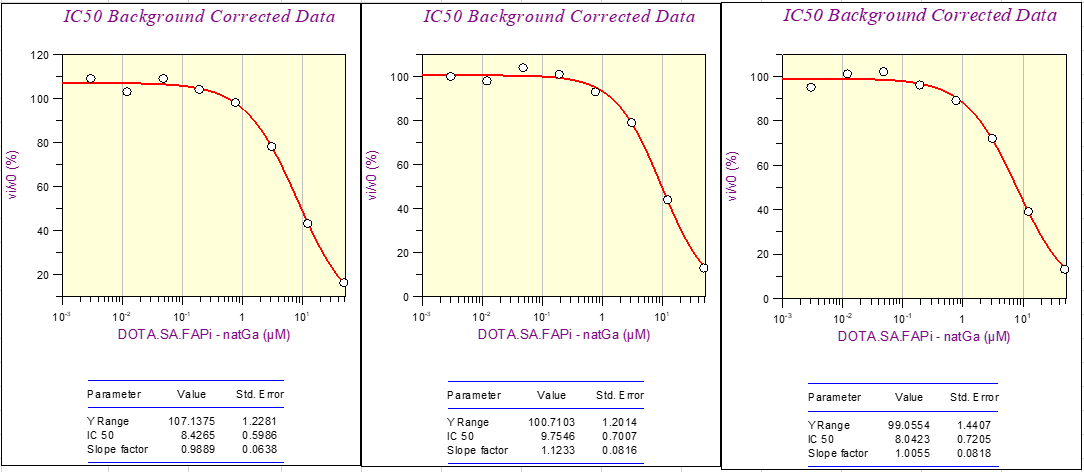


Figure S13: Inhibition assay graph and calculated IC_50_-data for ^nat^Ga-DOTA.SA.FAPi (n=3) with regard to PREP.


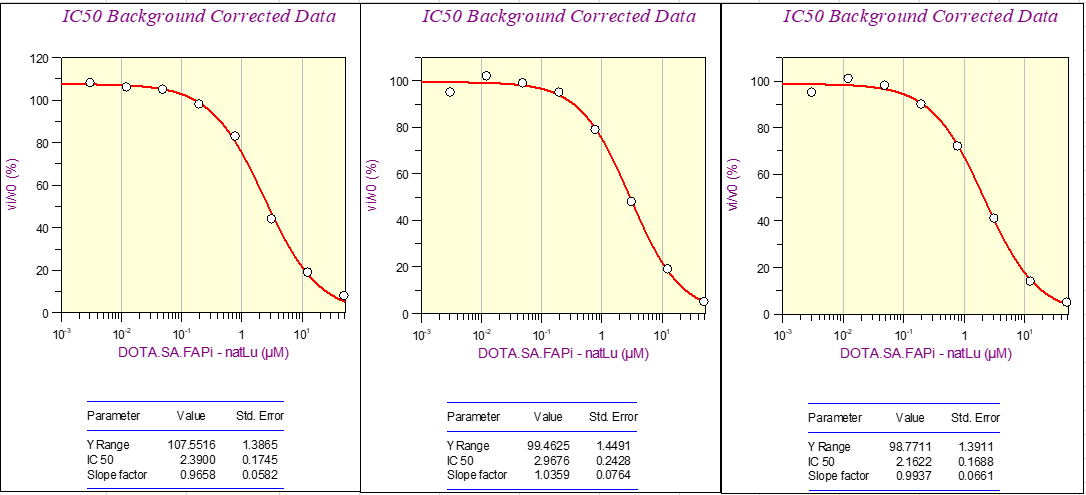


Figure S14: Inhibition assay graph and calculated IC_50_-data for ^nat^Lu-DOTA.SA.FAPi (n=3) with regard to PREP.

***
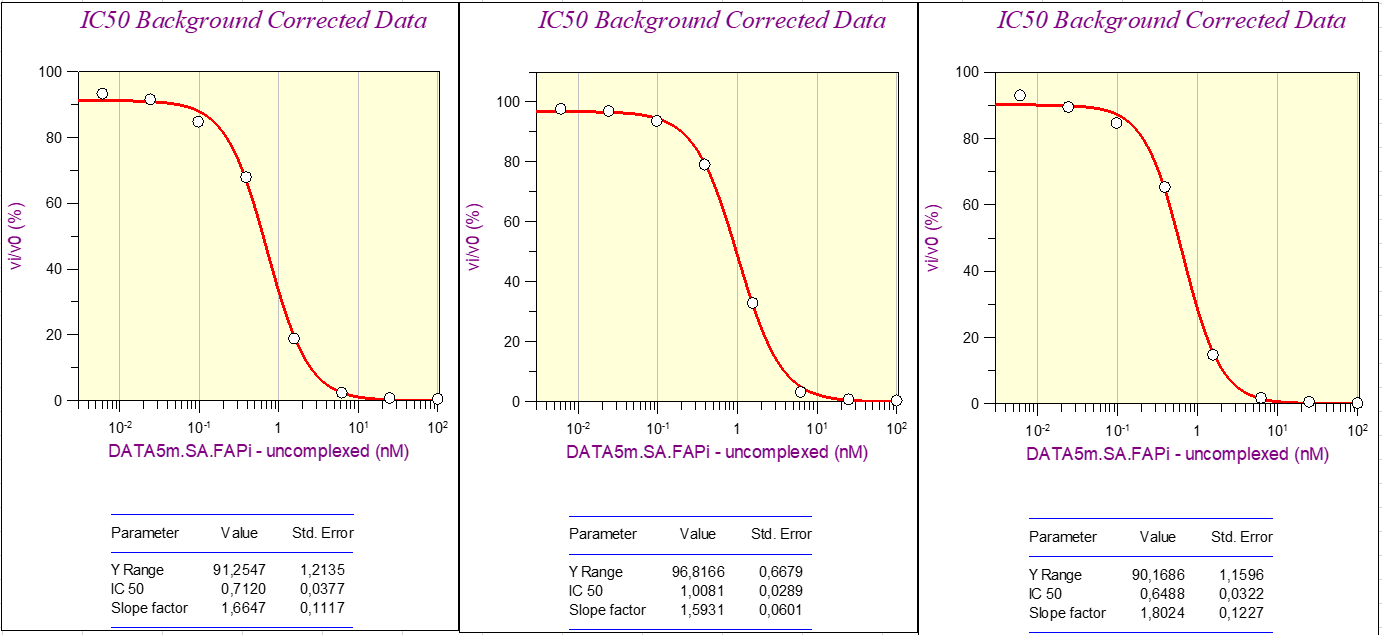
***

Figure S15: Inhibition assay graph and calculated IC_50_-data for DATA^5m^.SA.FAPi (n=3) with regard to FAP.

***
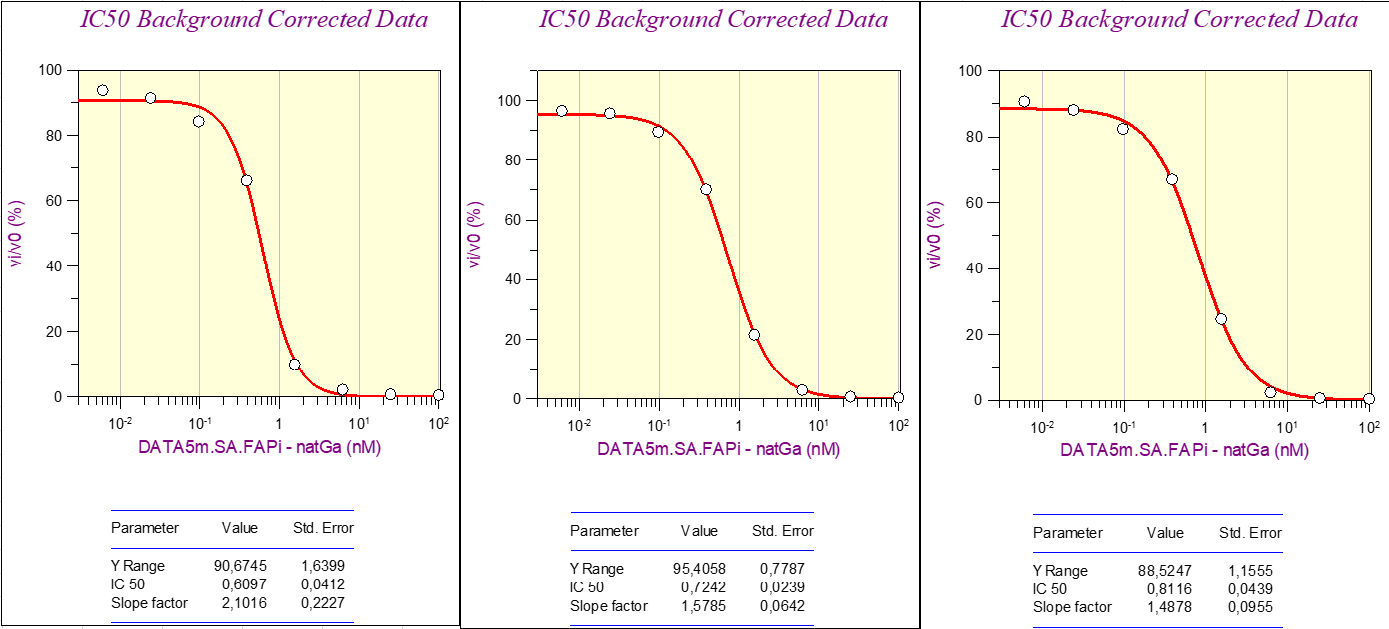
***

Figure S16: Inhibition assay graph and calculated IC_50_-data for ^nat^Ga- DATA^5m^.SA.FAPi (n=3) with regard to FAP.

***
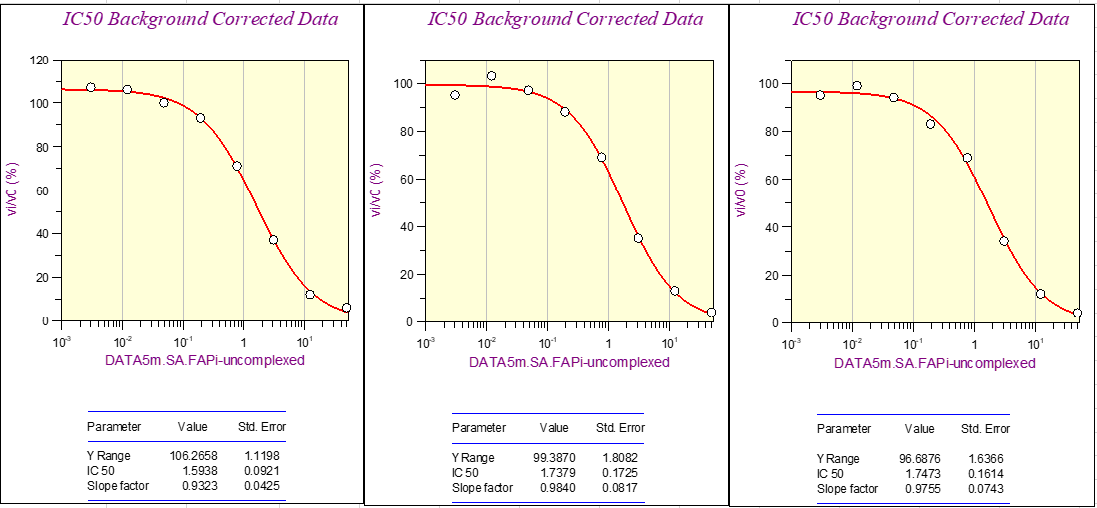
***

Figure S17: Inhibition assay graph and calculated IC_50_-data for DATA^5m^.SA.FAPi (n=3) with regard to PREP.

***
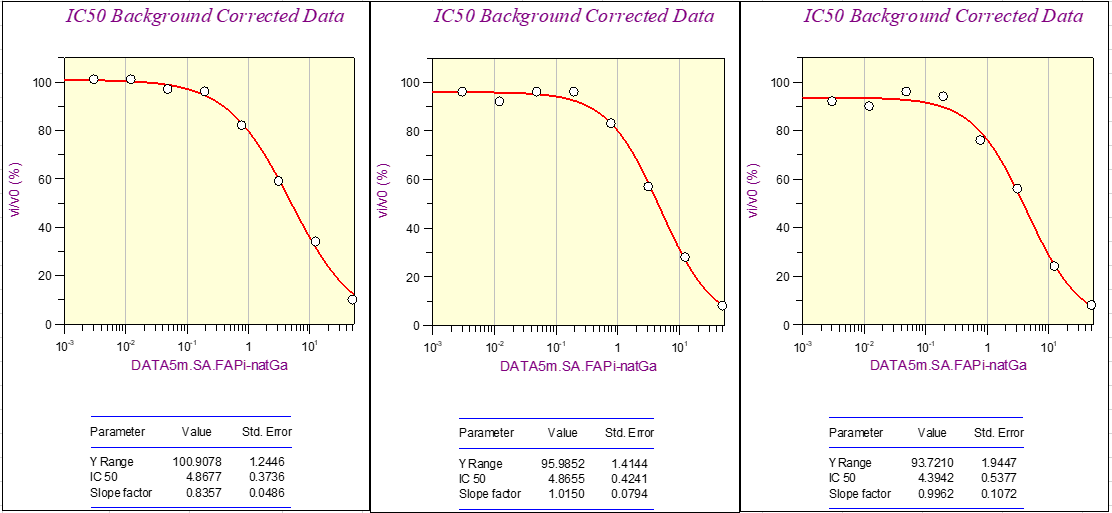
***

Figure S18: Inhibition assay graph and calculated IC_50_-data for ^nat^Ga-DATA^5m^.SA.FAPi (n=3) with regard to PREP.

***
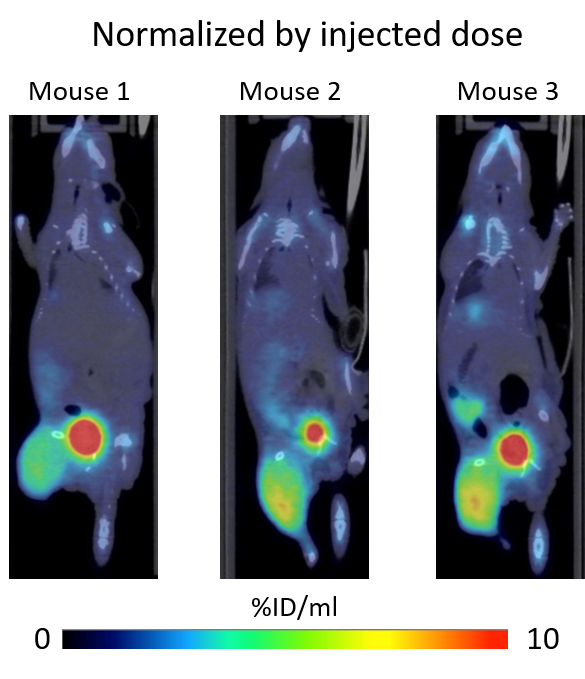
***

Figure S19: [^68^Ga]Ga-DOTA.SA.FAPi uptake in a HT-29 xenograft mouse model. Coronal 2D-fused PET/CT image (normalized by injected dose) 60 min after injection of [^68^Ga]Ga-DOTA.SA.FAPi.

***Ex vivo* biodistribution**

Table S1: *Ex vivo* biodistribution data of [^68^Ga]Ga-DOTA.SA.FAPi at 1 h p.i. (N=3)

| ***Ex vivo* biodistribution (in %ID/g)** | | |
| --- | --- | --- |
| **Organ** | **Average** | **SD** |
| Blood | 0.56 | 0.06 |
| Heart | 0.22 | 0.01 |
| Lungs | 0.33 | 0.04 |
| Liver | 0.70 | 0.19 |
| Spleen | 0.23 | 0.03 |
| Pancreas | 0.51 | 0.13 |
| Stomach | 0.21 | 0.04 |
| Small Intestine | 1.81 | 0.49 |
| Large Intestine | 0.21 | 0.01 |
| Kidneys | 1.77 | 0.37 |
| Bladder | 0.96 | 0.10 |
| Muscle | 0.45 | 0.09 |
| Fat | 0.38 | 0.29 |
| Bone | 3.56 | 0.34 |
| Skin | 0.04 | 0.00 |
| Brain | 1.27 | 0.50 |
| Tumor | 5.20 | 0.21 |
